# 16S rRNA gene sequencing on a benchtop sequencer: accuracy for identification of clinically important bacteria

**DOI:** 10.1111/jam.13590

**Published:** 2017-11-07

**Authors:** G.S. Watts, K. Youens‐Clark, M.J. Slepian, D.M. Wolk, M.M. Oshiro, G.S. Metzger, D. Dhingra, L.D. Cranmer, B.L. Hurwitz

**Affiliations:** ^1^ The University of Arizona Cancer Center University of Arizona Tucson AZ USA; ^2^ Department of Pharmacology University of Arizona Tucson AZ USA; ^3^ Department of Agricultural and Biosystems Engineering University of Arizona Tucson AZ USA; ^4^ Department of Medicine University of Arizona Tucson AZ USA; ^5^ Department of Biomedical Engineering University of Arizona Tucson AZ USA; ^6^ Arizona Center for Accelerated Biomedical Innovation University of Arizona Tucson AZ USA; ^7^ Pharmaceutical Sciences Geisinger Health System Danville PA USA; ^8^ Center for Infectious Disease Diagnostics and Research Wilkes University Geisinger Health System Danville PA USA; ^9^ Life Technologies Thermo Fisher Scientific Carlsbad CA USA; ^10^ School of Medicine Seattle WA USA; ^11^ University of Washington Fred Hutchinson Cancer Research Center and Seattle Cancer Care Alliance Seattle WA USA

**Keywords:** 16S rRNA gene, accuracy, bacterial identification, benchtop sequencer, sensitivity

## Abstract

**Aims:**

Test the choice of 16S rRNA gene amplicon and data analysis method on the accuracy of identification of clinically important bacteria utilizing a benchtop sequencer.

**Methods and Results:**

Nine 16S rRNA amplicons were tested on an Ion Torrent PGM to identify 41 strains of clinical importance. The V1–V2 region identified 40 of 41 isolates to the species level. Three data analysis methods were tested, finding that the Ribosomal Database Project's SequenceMatch outperformed BLAST and the Ion Reporter Metagenomics analysis pipeline. Lastly, 16S rRNA gene sequencing mixtures of four species through a six log range of dilution showed species were identifiable even when present as 0·1% of the mixture.

**Conclusions:**

Sequencing the V1–V2 16S rRNA gene region, made possible by the increased read length Ion Torrent PGM sequencer's 400 base pair chemistry, may be a better choice over other commonly used regions for identifying clinically important bacteria. In addition, the SequenceMatch algorithm, freely available from the Ribosomal Database Project, is a good choice for matching filtered reads to organisms. Lastly, 16S rRNA gene sequencing's sensitivity to the presence of a bacterial species at 0·1% of a mixture suggests it has sufficient sensitivity for samples in which important bacteria may be rare.

**Significance and Impact of the Study:**

We have validated 16S rRNA gene sequencing on a benchtop sequencer including simple mixtures of organisms; however, our results highlight deficits for clinical application in place of current identification methods.

## Introduction

When infection is suspected, timely and accurate identification of pathogens can improve patient outcomes and reduce the excessive use of broad‐spectrum antibiotic therapy. The current standard for infection diagnosis is based on culturing a sample followed by morphological and biochemical methods to identify isolates. Despite its widespread use and utility, culture‐based diagnosis of infection relies on outgrowth of one, or a few, dominant organisms over fastidious organisms present in the sample despite that slow, or nongrowing, organisms may be clinically important. This problem is exacerbated in chronic infections that are commonly multi‐organismal in nature (e.g. diabetic foot ulcers), when antibiotics are present, and when the causative organism is fastidious. The time required to obtain a culture‐based identification combined with the failure to culture fastidious organisms has resulted in the common practice of employing broad‐spectrum empiric antibiotic therapy with the assumption that the infection is bacterial, but sometimes resulting in nonoptimal treatment. Nonoptimal empiric therapy can increase negative outcomes, increase healthcare costs and contribute to the spread of antimicrobial resistance. For example, the problems of spectrum under‐coverage with empiric therapy were highlighted by Stoneking *et al*. who found 21% of the causative organisms in sepsis were not adequately covered by the empiric antibiotic administered, while a different antibiotic would have been given 37% of the time if a diagnosis had been available (Stoneking *et al*. 2013). Sequence‐based identification of bacteria utilizing 16S rRNA gene sequencing, which does not require culture, offers a way to circumvent the major drawbacks of culture‐based identification. 16S rRNA gene sequencing has been utilized for several decades as a supplement to culture‐based methods in clinical laboratories; however, utilizing 16S rRNA gene sequencing clinically has been hampered, in part, by the time and cost associated with sequencing. In addition, when the exact identification of organisms causing infection is critical, the parameters for successful utilization of 16S rRNA sequencing are different than those for broad microbiome surveys (reviewed in Clarridge 2004; Millar *et al*. 2007; Woo *et al*. 2008; Mancini *et al*. 2010). Lastly, 16S rRNA gene sequencing can be less sensitive and slower than pathogen‐specific polymerase chain reaction (PCR) assays using panels of primers. Nonetheless, 16S rRNA gene sequencing in the clinical setting has been of particular utility in instances where bacteria are unidentifiable by culture because they are fastidious, uncommon or biochemically inactive (Drancourt *et al*. 2000; Wilck *et al*. 2001; Fontana *et al*. 2005; Petti *et al*. 2005; Raoult *et al*. 2009; Woo *et al*. 2009; Salipante *et al*. 2013). While 16S rRNA gene sequencing has been successfully used clinically, drawbacks beyond time and cost have been noted, including lack of universality and the inability to differentiate between true pathogenic organisms, opportunists, and commensals (e.g. *Shigella* species versus *Escherichia coli*, respectively), sequencing errors, and intra‐species sequence heterogeneity (Ashelford *et al*. 2005; Boudewijns *et al*. 2006; Janda and Abbott 2007; Woo *et al*. 2007; Kunin *et al*. 2010).

16S rRNA gene sequencing for identification of bacteria on next‐generation sequencers involves PCR amplification of a region of the 16S rRNA gene followed by library preparation, sequencing and analysis of the sequence data. Sequence diversity, which provides the power to discriminate between bacterial species, varies across the 16S rRNA gene (Neefs *et al*. 1993; Ashelford *et al*. 2005). Nine 16S rRNA gene regions of high sequence variability have been identified (referred to as hypervariable regions V1–V9) and primers have been developed to amplify them (Baker *et al*. 2003; Wang and Qian 2009). The 16S rRNA hypervariable regions have varying lengths and conservation, and thus information density, resulting in differing ability to distinguish between bacteria (Chakravorty *et al*. 2007; Liu *et al*. 2007). In addition to evolutionary differences between species, 16S rRNA gene sequence can vary within the same genome since most species have multiple copies and between strains of the same species (Michon *et al*. 2012). Further sources of sequence diversity beyond biological variance include errors introduced by polymerases used in sequencing library preparation, platform‐specific sequencing error and the numerous choices made during data filtering and analysis, all of which can affect the accuracy of bacterial identification and can be difficult to isolate and control (Kunin *et al*. 2010; Degnan and Ochman 2012; Lee *et al*. 2012). Development of rapid, low‐cost benchtop sequencers, with their capability to provide same‐day results from a sample, has potentially increased the opportunity to utilize 16S rRNA gene sequencing for bacterial identification in clinical settings. Such modern sequencers could provide cost‐effective bacterial identification in less time than culture‐based diagnosis, while circumventing the problem of fastidious organisms. However, the new benchtop sequencers currently available (e.g. Ion Torrent PGM and Illumina MiSeq) cannot sequence the frequently utilized V1–V3 16S rRNA amplicon that covers the first third of the gene due to read length limitations, while those that can (e.g. Oxford Technologies MinION) currently have a prohibitively high error rate. Nonetheless, studies focused on clinically important organisms have utilized shorter hypervariable regions with the newer sequencers. For example, the Ion Torrent PGM was utilized to analyse organisms associated with periodontitis with the V6 hypervariable region of the 16S rRNA gene (Jünemann *et al*. 2012). More recently, a study compared the performance of the Illumina and Ion Torrent platforms when identifying clinically important bacteria using the V1–V2 16S rRNA hypervariable region finding both platforms had good concordance with a known set of organisms (Salipante *et al*. 2014).

Given all of the variables, we chose to examine the utilization of 16S rRNA gene sequencing on a modern sequencing platform. Rather than perform *in silico* experiments, we chose to start from live bacteria or DNA to capture all sources of error inherent to the approach. In this work, we sought to explore the sensitivity, accuracy and utility of 16S rRNA gene sequencing for identification of pure strains of clinically important bacteria utilizing the Ion Torrent Personal Genome Machine (PGM). We hypothesized that with the right platform, choice of target and amplicon, and data analysis method, 16S rRNA gene sequencing could provide the accuracy and sensitivity required for identification of clinically important bacteria across species and genera and across Gram‐positive and Gram‐negative organisms. To test our hypothesis, we used 41 strains to test the choice of 16S rRNA hypervariable region and data analysis method on accuracy of identification to the species level. Furthermore, we tested detection over a six log range of five composite mixtures of bacteria chosen to represent progressively finer and more difficult discriminations. Demonstrating the performance of 16S rRNA gene sequencing with a ‘short‐read’ platform such as the Ion Torrent PGM is useful for placing the approach in perspective with current options for bacterial identification and provides insight into next‐step approaches.

## Materials and methods

### V1, V3, V6, V1–V2 amplicons

The sequence and sizes of the sequencing templates for the V1, V3, V6 and V1–V2 16S rRNA gene hypervariable regions, including amplification primers, barcode adaptor and sequencing adaptors, are shown in Table 1. To determine the effect of hypervariable region on accuracy of identification, four 16S rRNA gene hypervariable regions were tested with 41 clinically important bacteria used for quality control strains for commercial assays and available as liquid cultures from the American Type Culture Collection (ATCC, Manassas, VA). Identities of the 41 strains analysed are shown in Table 2 along with their ATCC reference numbers. Five microlitres of liquid culture was used as PCR template to create V1, V3, V6 and V1–V2 amplicons with the AccuPrime *Taq* DNA Polymerase High Fidelity kit, the 10× AccuPrime PCR Buffer I (Thermo Scientific, Waltham, MA), and 300 nM primer concentration. Polymerase chain reaction conditions were: 95°C lysis per denaturation for 2 min; 29 cycles of 95°C denaturation for 20 s, 57°C annealing for 30 s and 72°C extension for 1 min; followed by a final extension step 72°C for 5 min. The 57°C PCR annealing temperature was determined by performing an initial temperature gradient experiment to determine the optimal annealing temperature for all four primer pairs. PCR products were purified using the Qiagen QIAquick PCR Purification Kit (Qiagen, Valencia, CA).

**Table 1:** Primer sequences and amplicon sizes for 16S rRNA gene hypervariable regions tested on 41 bacterial strains. bp, base pairs. All Primer sequences are from Sundquist *et al*. 2007; except for V6 forward primer which is from Wang and Qian 2009

Hypervariable region	Length of informative sequence (bp)	Length of sequencing template (bp)	Forward primer sequence	Reverse primer sequence
V1	72	196	AGAGTTTGATCMTGGCTCAG	TTACTCACCCGTICGCCRCT
V3	159	280	ACTCCTACGGGAGGCAGCAG	ATTACCGCGGCTGCTGG
V6	68	189	ACGCGARGAACCTTACC	ACGAGCTGACGACARCCATG
V1–V2	313	437	AGAGTTTGATCMTGGCTCAG	CYIACTGCTGCCTCCCGTAG

**Table 2:** Identities of the 41 strains used to test performance of hypervariable regions and analysis methods. The strains utilized were chosen because of their use as quality control strains for commercial assays available through the American Type Culture Collection

ATCC number	Organism name
ATCC 12453	*Proteus mirabilis* Hauser
BAA 1721	*Staphylococcus aureus* subsp. aureus Rosenbach
BAA 1720	*Staphylococcus aureus* subsp. aureus Rosenbach
ATCC 15305	*Staphylococcus saprophyticus*
ATCC 35984	*Staphylococcus epidermidis*
ATCC 51299	*Enterococcus faecalis*
ATCC 29212	*Enterococcus faecalis*
ATCC 12228	*Staphylococcus epidermidis*
ATCC 43300	*Staphylococcus aureus*, methicillin resistant
ATCC 51559	*Enterococcus faecium*
ATCC 6305	*Streptococcus pneumoniae*
BAA 752	*Kocuria kristinae*
ATCC 25922	*Escherichia coli*
ATCC 19615	*Streptococcus pyogenes*
ATCC 19258	*Streptococcus salivarius* (*thermophilus*)
ATCC 43079	*Streptococcus equi*
ATCC 43076	*Enterococcus saccharolyticus*
ATCC 700327	*Enterococcus casseliflavus*
BAA 750	*Staphylococcus saprophyticus*
BAA 751	*Listeria monocytogenes*
ATCC 49619	*Streptococcus pneumoniae*
ATCC 29061	*Staphylococcus sciuri*
ATCC 700324	*Klebsiella oxytoca*
ATCC 17666	*Stenotrophomonas maltophilia*
ATCC 13253	*Elizabethkingia meningoseptica*
BAA 1293	*Corynebacterium striatum*
ATCC 7070	*Paenibacillus polymyxa*
ATCC 43534	*Oligella ureolytica*
ATCC 27853	*Pseudomonas aeruginosa*
ATCC 25931	*Shigella sonnei*
BAA 749	*Ochrobactrum anthropi*
BAA 751	*Listeria monocytogenes*
ATCC 6380	*Proteus vulgaris*
ATCC 1296	*Bacteroides ovatus*
ATCC 9714	*Clostridium sordellii*
BAA 1152	*Eikenella corrodens*
ATCC 13124	*Clostridium perfringens*
ATCC 12464	*Clostridium septicum*
ATCC 33389	*Aggregatibacter aphrophilus*
ATCC 9007	*Haemophilus influenzae*
ATCC 19424	*Neisseria gonorrhoeae*

### 16S rRNA metagenomics kit sequencing

We further tested the choice of hypervariable region on accuracy utilizing the Ion 16S Metagenomics kit (Thermo Fisher, Waltham, MA) in a subset of 11 of the 41 strains used to test the V1, V3, V6 and V1–V2 regions. The 11 strains were selected to represent those that were poorly characterized by the V1, V3 and V6 amplicons to test if the additional hypervariable regions in the kit allowed more accurate identification. Amplicons from the V2, V3, V4, V6–V7, V8 and V9 hypervariable region of the 16S rRNA gene were generated using the Ion 16S Metagenomics kit following the manufacturer's protocol. Five nanograms of bacterial DNA was amplified for each of the 11 strains chosen for testing and barcoded libraries created as described in the Metagenomics kit protocol. Equimolar amounts of each library were used to seed an Ion OneTouch OT2 400 base pair reaction and enriched using the Ion OneTouch ES system. Enriched, templated beads were loaded onto an Ion Torrent 318 V2 chip and sequenced using the Ion PGM Sequencing 400 kit.

### 16S rRNA gene sequencing

V1, V3, V6 amplicons: Life Technologies (Carlsbad, CA, USA) components for Ion Torrent sequencing were utilized in the following manner: amplicon libraries were prepared using the Ion Plus Fragment Library Kit and molecularly barcoded with IonXpress Barcode Adapters. Polymerase chain reaction amplicons from V1, V3 and V6 16S rRNA PCR reactions using all 41 strains were seeded into the library preparation kit reaction at a ratio of 1:1:3 (V1:V6:V3) to adjust for the competitive inhibition against the V3 amplicon due to its length, which is approximately twice that of V1 and V6. Barcoded libraries were quantified using the Ion Library Quantitation kit, and equimolar amounts of library were added to an Ion OneTouch 200 V2 DL reaction. The resulting templated beads enriched with the Ion OneTouch ES system were quantitated with a Qubit Ion Sphere Quality Control kit (Life Technologies) on a Qubit 3.0 fluorimeter (Qubit, New York, NY). Enriched templated beads were loaded onto Ion Torrent 314 V2 chips and sequenced according to the manufacturer's protocol using the Ion PGM sequencing 200 kit.

V1–V2 amplicon: V1–V2 sequencing libraries were prepared as described above; however, sequencing was performed with the Ion PGM sequencing 400 kit.

Bacterial Mixture V1–V2 amplicons: Mixtures were created from DNA or bacterial cultures, both purchased from the ATCC (Table 3, and described below). Template preparation and sequencing of the libraries of bacterial mixtures were as follows: equimolar amounts of each barcoded amplicon were pooled and used to seed an Ion PGM Template OT2 400 reaction. Enriched templated beads were loaded onto an Ion Torrent 318 V2 chip and sequenced using the Ion PGM Sequencing 400 kit.

**Table 3:** Identities of bacteria used to create binary mixtures, and composition of those mixtures. Mixtures were used to test sensitivity of 16S rRNA V1–V2 sequencing in a six log range of ratios

Organism	ATCC catalogue number
*Staphylococcus aureus,* methicillin sensitive (MSSA)	BAA‐1718D‐5
*Staphylococcus aureus,* methicillin resistant (MRSA)	BAA‐1717D‐5
*Escherichia coli*	25922, 25922D‐5
*Shigella sonnei*	29903D‐5
*Staphylococcus saprophyticus*	15305, 15305D‐5
*Streptococcus pyogenes*	12344D‐5

### 16S rRNA sequence data analysis

To test the effect of data analysis method on accuracy, we compared the identifications made by three available algorithms using the same V1–V2 hypervariable region sequence data as follows:

Ion Reporter metagenomics workflow: Unaligned binary data files (Binary Alignment Map, BAM) generated by the Ion Torrent PGM were uploaded to Ion Reporter (https://ionreporter.thermofisher.com/ir/) and analysed using default settings. Calls were made by assessing the most specific unique taxonomic level identified using the mapped sequences. For example, if two genera in the same family were identified using the sequence for a particular strain, the result was a family‐level call.

blast‐based analysis: Unaligned BAM files were converted to FASTQ format and passed to cutadapt ver. 1.2.1 (https://cutadapt.readthedocs.org/en/stable/) for removal of primer sequences, quality trimming and length filtering. Because some primers used to generate the 16S rRNA gene amplicons contained redundant nucleotides (Table 1), all possible primer sequences were provided to cutadapt when relevant. Primer sequence removal was performed using default cutadapt settings with quality trimming by Phred quality score set to 20, and length filtering set to remove any read whose length was less than 75% of the expected full‐length amplicon in question (e.g. V1–V2 reads less than 234 base pairs in length were removed, expected sizes for amplicons are shown in column 2 of Table 1). Resulting FASTQ files were converted to FASTA format and used as input to blastn (ncbi‐blast‐2.2.29+; Camacho *et al*. 2009) utilizing a 16S Microbial database downloaded from the National Center Biotechnology Information (ftp://ftp.ncbi.nlm.nih.gov/blast/db). To be included in the results, a match had to have greater than 98% identity with the query and an e‐value less than 1e‐50. Results from the blastn algorithm were parsed to keep only the first best match based on bitscore. The number of matches to every organism detected within each sample were counted and used to determine the taxonomic level reached by comparing the most prevalent organism to the correct identity.

Ribosomal Database Project (RDP): The FASTA files generated for blastn analysis above were analysed utilizing the SequenceMatch algorithm in the RDP Tools suite (https://github.com/rdpstaff/RDPTools) with the number of matches set to one for each sequence. A reference file was created for use by the SequenceMatch algorithm by downloading sequences from the RDP database using the Hierarchy Browser. Sequences were selected with the strain option set to ‘type’ and ‘nontype’, the source set to ‘isolates’, the size set to ‘>1200’, and the quality set to ‘good’. The resulting reference FASTA file was further edited to remove entries with nontaxonomic headers (e.g. ‘marine bacterium’, ‘arsenic‐oxidizing’). Results for each read analysed were annotated with the organism name, and the number of matches to each organism detected within each sample was counted and used to determine the taxonomic level reached by comparing the most prevalent organism to the correct identity.

### Binary mixtures of bacteria

To investigate the ability of 16S rRNA gene sequencing to detect bacteria when present at varying concentrations in mixtures, we employed two strategies to create binary mixtures: (i) mixing purified DNA and (ii) mixing live bacteria followed by DNA isolation. Mixtures were made to cover six logs of dilution as follows: Bacterial DNA isolated from methicillin‐sensitive *Staphylococcus aureus* (MSSA, ATCC BAA‐1718D‐5), methicillin‐resistant *S. aureus* (MRSA, ATCC BAA‐1717D‐5), *Staphylococcus saprophyticus* (15305D‐5), *Shigella flexneri* (ATCC 29903D‐5), *Streptococcus pyogenes* (ATCC 12344D‐5) and *Escherichia coli* (ATCC 25922D‐5) was purchased from the ATCC. Five pairs of mixtures were made with the pairs chosen to represent: (i) strains with differing antibiotic sensitivity patterns, which could be clinically significant were competitive inhibition to occur and mask the presence of a drug‐resistant strain (e.g. MSSA versus MRSA); (ii) difficult to discriminate species with divergent clinical impact (*E. coli* versus *S. flexneri*); (iii) Gram‐positive species (*S. saprophyticus* versus *S. pyogenes*), and Gram‐positive versus Gram‐negative species (*E. coli* versus *S. saprophyticus*). DNA was resuspended in sterile phosphate buffered saline, quantitated from absorption at 260 nm utilizing a NanoDrop ND‐1000 spectrophotometer, and used to create binary mixtures of the following ratios by mass: 0·1:99·9, 1:99, 10:90, 50:50, 90:10, 99:1, 99·9:0·1. DNA mixtures were diluted to a final concentration of 0·1 ng μl^−1^ and used as DNA template to generate 16S rRNA V1–V2 PCR amplicons. Polymerase chain reaction was performed using 2·5 μl of the bacterial DNA mix (250 pg total) in an Amplitaq Gold 360 Master Mix reaction (Thermo Scientific), using the supplied GC enhancer and 300 nM primer concentration. Polymerase chain reaction conditions were as follows: 95°C for 10 min; 16 cycles of 95°C denaturation for 30 s, 57°C annealing for 30 s, 72°C extension for 30 s; followed by a final 7‐min extension at 72°C. Polymerase chain reaction amplicons were purified using the Agencourt AMPure XP beads (Beckman Coulter, Indianapolis, IN) according to the manufacturer's protocol; yields ranged from 4 to 20 ng.

For the live bacteria mixture, freeze‐dried bacterial pellets of *E. coli* (ATCC 25922) and *S. saprophyticus* (ATCC 15305) were purchased from ATCC. The pellets were re‐resuspended in 5 mm of the recommended culture media and grown overnight at 37°C with orbital shaking at 180 rev per min^−1^ and stored at 4°C. Bacterial density was determined by averaging triplicate optical density measurements at a wavelength of 600 nm on a NanoDrop ND‐1000 spectrophotometer. *Escherichia coli* and *S. saprophyticus* were mixed in the same ratios as the bacterial DNA described above, to an equivalent total density of 2 × 10^8^ bacteria per ml. Genomic DNA was isolated from the bacterial mixtures using the Qiagen DNeasy Blood and Tissue Kit (Qiagen, Valencia, CA) utilizing the Gram‐positive pretreatment protocol and the optional RNase treatment step. DNA concentration was determined from absorption at 260 nm with a Nanodrop ND‐1000, followed by dilution to 0·1 ng μl^−1^. 2·5 μl of diluted DNA used as PCR template as described above. Sequencing was performed as described for the 41 strains, while data analysis was performed utilizing the RDP SequenceMatch algorithm as described above.

## Results

### Identification of 41 strains using 16S rRNA hypervariable regions V1, V3, V6

Current benchtop sequencers with sufficient read accuracy cannot sequence the full length of the 16S rRNA gene, and therefore, three commonly used hypervariable regions (V1, V3, V6) were selected to test the accuracy of identification of 41 clinically important strains. By analysing strains of known identity, we were able to assess the effect of hypervariable regions and analysis method on accuracy. Despite the size of the V3 amplicon exceeding Ion Torrent's 200 base pair sequencing specification, full‐length reads were obtained (data not shown). Following amplicon generation, and sequencing, an identification was made for each hypervariable region/strain combination utilizing the RDP SequenceMatch algorithm. The median number of reads used for identification of the 41 strains following all filtering steps was 2 040 (range 450–14 126), 5,704 (range 338–25 852) and 1 008 (196–12 310) for V1, V3 and V6, respectively.

Results are summarized in Table 4 which shows that none of the three regions tested were able to identify more than 21 of the 41 strains to the species level. The V1 hypervariable region with 21 species‐level identifications performed best, despite having shorter sequence length than V3. While the V6 region only made 11 species calls with multiple calls above the genus level, indicating it is particularly unsuited to clinical applications. The taxonomic level reached by each of the hypervariable regions is shown in Table S1, which reveals that, in particular, members of the Enterobacteriales and Lactobacillales orders were not accurately identified.

**Table 4:** Taxonomic level reached when identifying 41 bacterial strains using four 16S rRNA hypervariable regions. Number of species identified at each taxonomic level are shown

Taxonomic level	16S rRNA Hypervariable region			
	V1	V3	V6	V1–V2
Kingdom	0	0	**3**	0
Phylum	0	0	0	0
Class	0	0	**7**	0
Order	**2**	**5**	**2**	0
Family	**6**	**6**	**7**	0
Genus	**10**	**10**	**11**	**1**
Species	**21**	**20**	**11**	**40**
No call	**2**	0	**0**	0

Bold was used to visually distinguish the values from those that are zero.

### Identification of 41 strains using 16S rRNA hypervariable region V1–V2

When 400 base pair sequencing chemistry became available for the Ion Torrent PGM, utilizing longer 16S rRNA amplicons containing more than one hypervariable region became feasible. While the V1–V3 amplicon which has been used in the past remained too long, the shorter V1–V2 amplicon, with a total sequencing template length of approximately 437 base pairs, was chosen for testing. Amplicons representing the 16S rRNA V1–V2 hypervariable region were generated for all 41 strains and analysed utilizing RDP's SequenceMatch algorithm. The number of reads analysed after quality and length filtering ranged from 16 744 to 301 524 with a median of 116 670. When used to identify the 41 strains, the V1–V2 region was more accurate than the shorter V1, V3, and V6 amplicons, allowing 40 (98%) of the strains to be identified to the species level, with one strain identified at the genus level (Table 4). The sole genus‐level call using V1–V2 sequence was for *Kocuria kristinae* which was misidentified as *Kocuria rosae* (Table S1). Of note, several difficult to differentiate species were correctly identified, including *Escherichia coli*,* Shigella sonnei*,* Enterococcus* species and *Proteus vulgaris*. Finally, the taxonomic level reached by the V1–V2 hypervariable region contained greater than 95% of all reads analysed except for three of the 41 strains: *Enterococcus casseliflavus*,* Shigella sonnei* and *Proteus vulgaris* (Table S1).

### Accuracy of additional hypervariable regions utilizing the Ion Torrent metagenomics kit

After the testing described above was complete, Ion Torrent released a metagenomics kit that utilizes the V2, V3, V4, V6–V7, V8 and V9 regions, all but one of which we had not previously tested. Eleven of the 41 strains were selected to test the Ion Torrent Metagenomics kit. Sequence reads for the Metagenomics kit amplicons were analysed using the Ion Reporter Metagenomics workflow and results summarized in Table S2. Of the 66 calls (6 regions in 11 strains), there were 35 family‐level, 20 genus‐level, and two species‐level calls across all regions with nine no calls due to lack of sequence. None of the hypervariable regions tested proved superior to the V1–V2 hypervariable region described above. A feature of the Ion Reporter Metagenomics workflow is the ability to generate a consensus call using the combined information from all regions sequenced. As shown in Table S2, the consensus call achieved only genus‐level calls with a low of 47% of reads assigned to genus level for *Ochrobactrum anthropi* and a high of 94% for *S. aureus* and *Staphylococcus epidermidis*.

### Role of analysis algorithm in identifying bacteria from 16S rRNA sequence

Numerous choices are made between the acquisition of a raw sequence file and making an identification from the reads contained therein, including trimming primer sequences, Phred quality score filtering or trimming, filtering on read length, summarizing read information and choice of reference database. To test the impact of the last two choices, we compared the results of two freely available methods: NCBI's blastn and RDP's SequenceMatch, and the commercially available Ion Torrent Metagenomics workflow utilizing the V1–V2 data generated from the 41 known strains described above. For the first two methods, following primer removal, quality trimming and read length filtering, sequence reads were passed to the algorithms in FASTA format and the best match (bitscore for blastn, similarity score for SequenceMatch) identified. Identified species were counted for each sample, and the most prevalent organism used to determine the taxonomic level reached. The Ion Reporter Metagenomics workflow was created to process Ion Torrent data generated with Ion 16S Metagenomics Kit which does not contain primers for the V1–V2 region. However, the workflow can be modified to analyse data from any 16S rRNA region, thus allowing analysis of the data we generated from the V1–V2 hypervariable region of the 41 strains.

Results from the three analysis methods are summarized in Table 5, revealing that the RDP SequenceMatch algorithm with a curated reference (see Materials and methods) performed the best, identifying 40 of 41 strains to the species level with one genus‐level call. The single failure of RDP SequenceMatch was in identification of *Kocuria kristinae* as *Kocuria rosea*. The two species have highly similar sequence in the 16S rRNA V1–V2 region, which resulted in equally good matches to both species in the SequenceMatch algorithm. Our approach selected the nearest neighbour match (*k* = 1), which happened to be *K. rosae*, despite *K. kristinae* matching equally well, and thus the incorrect organism was reported most frequently.

**Table 5:** Taxonomic level reached for 41 bacterial strains using the Ribosomal Database Project SequenceMatch (RDP), NCBI blastn (BLAST), and Ion Torrent's Ion Reporter Metagenomics workflow (IR) algorithms for 41 bacterial strains

Taxonomic level	Analysis method		
	RDP	BLAST	IR
Kingdom	0	0	0
Phylum	0	0	0
Class	0	0	0
Order	0	0	0
Family	0	**1**	**3**
Genus	**1**	**1**	**9**
Species	**40**	**39**	**29**

Bold was used to visually distinguish the values from those that are zero.

The accuracy of the RDP SequenceMatch algorithm was closely followed by blastn using the NCBI 16S Microbial database, which identified 39 of 41 strains to the species level with one genus‐level call (*Corynebacterium striatum* misidentified as *Corynebacterium simulans*) and one family‐level call (*E. coli* misidentified as *S. dysenteriae*). In addition, the blastn algorithm reported a nearly equal number of matches to *Haemophilus aegyptius* as to the correct species *Haemophilus influenzae* (16 961 vs 17 605 respectively, Table S3). Both *Haemophilus* species have highly similar 16S rRNA sequences, and examination of the sequence match to *H. aegyptius* revealed divergence from *H. influenzae* in areas of the V1–V2 sequence that would be expected to contain platform‐specific sequence errors (data not shown). The blastn algorithm did not encounter the same issue with discriminating *K. kristinae* from *K. rosae* due to the fact that the *K. rosae* reference sequence that was matched in SequenceMatch was not present in the blastn database.

The Ion Reporter metagenomics workflow identified 29 of 41 strains to the species level, nine to the genus level and three to the family level. The Ion Reporter Metagenomics workflow reports results for all reads as a percent of total, as shown in Table S3. For every strain analysed, >98% of reads matched the family, genus or species called, with the nonmatching species providing insight into two sources of error. The first source of error was due to closely related species whose sequence was indistinguishable from the correct species resulting in the nine genus and three family calls (e.g. *S. epidermidis* was not be differentiated from *Staphylococcus equi*). Similarly, the *Enterobacteriaceae* containing *E. coli* and *S. sonnei* accounted for two of the three family‐level calls, with *Streptococcus pyogenes* the third. The second apparent source of error was low numbers of reads from adjacent organisms in Table S3, suggesting cross‐over contamination from adjacent wells during sequencing library preparation (e.g. 0·07% of reads in the *Listeria monocytogenes* sample were identified as belonging to the genus of the adjacent *O. anthropi* sample). None of the contaminating organism's calls accounted for more than 2% of reads mapped, with all but one under 1% (Table S3). Similar results were found for nonmatching organisms called by the SequenceMatch and blastn algorithms, in which reads were found in every sample that matched either: (i) closely related strains representing misidentification due to sequence generation error or natural sequence heterogeneity; or (ii) other strains used in the experiment, suggesting cross‐over contamination during either sample handling, library preparation or sequencing. When the first type of error was prevalent (e.g. *Haemophilus influenzae* was frequently misidentified as *Haemophilus aegyptius* by blastn, Table S4), the percent of hits not matching the correct call reached 49% of total, and when low, the error rates were typically less than 2% of reads (Table S3). Of note, the RDP SequenceMatch and NCBI blastn algorithms produced disparate error rates for the same organisms. For example, SequenceMatch had a 38·6% error rate for *Enterococcus casseliflavus* due to frequent misidentification as *Enterococcus gallinarum*, while blastn's error rate for this strain was only 1%. Conversely, blastn had a 39% error rate for *E. coli*, while SequenceMatch's error rate was 1·7%.

### Sensitivity of 16S rRNA V1–V2 sequencing to binary mixtures of related bacterial species

**Figure 1:**
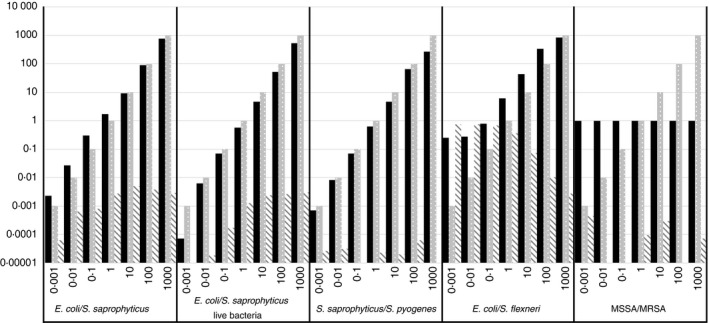
16S rRNA gene sequencing sensitivity and accuracy. Expected (grey bars) versus observed (black bars) ratios for binary mixtures of DNA and live bacteria ranging from 0·1 to 99·9% for each organism are graphed. Error ratio (striped bars), total count of species identified that were not added to the mixture divided by total number of reads. ■ Observed ratio; 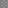 Expected ratio; 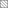 Error ratio.

Having established the V1–V2 hypervariable region and RDP's SequenceMatch as the best combination analysed, we sought to test the linearity and threshold for detection with controlled mixtures of two bacterial species. The number of reads that passed all filtering steps for all 35 samples generated from the mixtures ranged from 11 044 to 66 555 with a median of 40 282. The number of reads matching the mixed organisms were used to calculate a ratio for comparison to the actual mixing ratio and graphed in Fig. 1. In addition, a measure of the error from misidentification and cross‐contamination was calculated by comparing the number of reads from any organism not matching the two that were mixed relative to the total number of reads analysed. 16S rRNA V1–V2 gene sequencing was able to detect the minority organism at both ends of the dilution series (0·1:99·9 and 99·9:0·1) in the mixtures of *E. coli*/*S. saprophyticus* (DNA and live bacteria) and *S. saprophyticus*/*S. pyogenes*. In addition, the dilution series showed a log‐linear response. In the first two mixtures containing *E. coli*, error was highest due to misidentification of *E. coli* reads as other members of the Enterobacteriaceae family. Even so, the number of erroneous calls never rose above 1% of the total number of reads. When *E. coli* was not present in the mixture, as in the *S. saprophyticus/S. pyogenes* dilutions, the error ratio fell below 0·01% and was zero in two dilutions. In contrast, the *E. coli*/*S. flexneri* mixture shows less linearity, due primarily to a higher rate of misidentification of *S. flexneri* reads as other members of the Enterobacteriaceae family, indicated by the decreasing error ratio as the amount of *S. flexneri* decreased. Finally, V1–V2 reads failed to separate MSSA from MRSA in any ratio, with all reads identified as *S. aureus*, and resulting in the observed ratio equalling one in every dilution.

## Discussion

16S rRNA gene sequencing offers potential advantages over culture‐based assays, including those based on mass spectrometry, mostly derived from the fact that it can be performed without culture and is thus free of issues such as outgrowth of fast‐growing versus fastidious organisms. However, until recently, the cost and time required to perform sequencing offset the advantages conferred by culture‐less analysis. Sequencers such as the Ion Torrent PGM and Illumina MiSeq offer the possibility of same‐day results, removing a significant barrier to adoption of 16S rRNA gene sequencing. Given the potential for improving the management of infections through rapid identification of pathogens utilizing benchtop sequencers, we sought to determine the 16S rRNA region and analysis method that would provide accurate identification of clinically important bacteria and test the approach's sensitivity. While 16S rRNA gene sequencing has been used extensively in surveys of diverse complex microbial communities (Kysela *et al*. 2005; Dethlefsen *et al*. 2008; Whiteley *et al*. 2012), the needs for clinical diagnosis of infections from normally sterile sites (i.e. low complexity samples containing one to a few dozen organisms) are different. Classification to the taxonomic level of order or family is adequate, or even desirable, in surveys of complex bacterial communities. In contrast, optimal antibiotic choices based on predicted resistance, infection source control, prediction of bacterial contaminants in normally sterile cultures and epidemiological tracking all benefit from exact identifications. To this end, we tested several 16S rRNA hypervariable regions within the read length limitations of modern sequencers. Of the amplicons tested, the V1–V2 amplicon performed better than any the others, with approximately twice as many species‐level calls (40 vs 21, 20 and 11 for V1, V3 and V6, respectively) than the next best region (V1). The failure to achieve at least a species‐level identification of *E. coli* was not unexpected, with the known difficulty in differentiating *E. coli* from *Shigella* sp. previously identified for the V1–V2 region (Salipante *et al*. 2014). While members of the *Streptococcus* and *Staphylococcus* genera were clustered together by Salipante *et al*. in their analysis of the V1–V2 hypervariable region on Illumina and Ion Torrent platforms, all species of these two genera were identified to the species level by the V1–V2 region in our study, regardless of analysis method used. The discrepancy between the results of Salipante *et al*. and our results for these two genera could be a result of data filtering and analysis choices when handling 16S rRNA sequence data or the sample sources. None of the amplicons targeted by the Ion Torrent Metagenomics kit primers improved upon the results achieved by the V1–V2 hypervariable region. Of note, combining the results of all the regions included in the kit to achieve a consensus identification did not improve performance, indicating that the information density of individual hypervariable regions sequenced separately is not additive.

Potential difficulties in utilizing 16S rRNA gene sequencing have long been recognized (Wintzingerode *et al*. 1997), including intra‐genomic variation between the copies within a bacteria's genome (Coenye and Vandamme 2003; Marchandin *et al*. 2003; Acinas *et al*. 2004), genetic drift between strains, errors introduced by polymerase enzymes during amplification (Schloss *et al*. 2011), sequencing error (Schloss *et al*. 2011) and differences in analytical technique (Schloss and Westcott 2011). The laboratory that seeks to adopt 16S rRNA gene sequencing can spend considerable effort investigating, suppressing and correcting for potential errors, but ultimately, their sum effect must be assessed with respect to their effect on accurate identification. This was the primary motivation for performing *in vitro* experiments employing live, pure strains of bacteria and DNA, rather than performing *in silico* experiments using sequence in publically available databases that may or may not accurately mirror the errors specific to the technology platform, reagents and our laboratory. Indeed, our results utilizing SequenceMatch and blastn demonstrated scenarios in which misidentifications due to database content and sequencing error can occur when species have highly similar 16S rRNA sequences. In the case of *K. kristinae*, the SequenceMatch reference sequences contained a sequence of *K. rosea* (GenBank Accession EF522129.1) that was matched equally well by the algorithm to sequence from *K. kristinae*. In contrast, the EF522129.1 reference sequence is not present in the blastn database, and thus, the blastn algorithm consistently made the correct identification. However, it should be noted that the correct identification in this case is likely a genus‐level identification since the species are so similar in sequence. Interestingly, when the SequenceMatch reference sequence EF522129.1 is used as a blastn query, the best match is still *K. kristinae*. This discrepancy may indicate that the sample was misidentified when it was sequenced and entered into GenBank, or simply be the result of differences in the way the two algorithms compare sequences. Another example was the discrepancy between the algorithms identification of *H. influenzae* which can be argued to result from sequencing error as the divergence from reference occurred in regions that would be prone to platform‐specific homopolymer error (Kunin *et al*. 2010). These two examples along with the other misidentifications of *E. coli* and *C. striatum* by blastn ultimately reflect the limitations of the 16S rRNA approach as sequence diversity between species approaches zero. Our approach of selecting the top match makes sense in the context of our study in which the true identity was known, but in actual clinical practice, identifications must be broader to reflect the uncertainties created by the combination of highly similar sequences in different species and sequencing error. These limitations are reflected in the recommendations found in clinical guidelines (CLSI, 2008) which require varying percent identities between organisms to make positive identifications. Despite these limitations, our results support a conclusion that the V1–V2 hypervariable 16S rRNA gene region paired with the RDP SequenceMatch algorithm can perform well within the limits of a modern benchtop sequencer, with 98% of species analysed correctly identified, and sensitivity to detect organisms down to 0·1% of a mixture. The accuracy achieved by our approach is similar to that attained by phenotypic and proteomic methods. Although we did not specifically test the effect of background DNA derived from a host (e.g. DNA derived from white cells in a blood sample) on sensitivity, our utilization of pure samples (either liquid cultures from pure strains or isolated DNA) in our experiments is analogous to the use of culture isolates in other bacterial identification approaches. Of the three analytical methods tested, both blastn and SequenceMatch are available for no cost as both website applications and as packages for local installation and use, and have been peer reviewed and utilized widely. In contrast, the Ion Reporter Metagenomics workflow is available through the Ion Reporter website which has costs associated with data storage and analysis. While there are costs associated with it, the Metagenomics workflow can provide consensus analysis of multiple hypervariable regions and has a more user‐friendly interface than the other two. In addition, the Ion Reporter site allows sample analysis tracking, cloud‐based storage of results and commentary and approval options that could be of use to a clinical laboratory.

While cross‐contamination was evident in all experiments, it did not cause errors in identification. Rather, the failures to reach species‐level identifications were derived from lack of sequence variability inherent to particular species, not technical concerns such as sequencing technology or data processing. Nevertheless, scrupulous amplicon control practices, unidirectional workflow and engineered laboratory space would be required in a clinical setting to minimize the contamination we observed. Examination of the identity of species that did not match the actual strain analysed provided information about the nature of the miscalls (Tables S3 and S4). In the great majority of cases, the miscalled organism was in the sample set analysed, suggesting that contamination was from concurrently analysed samples and not sources such as personnel or reagents in the laboratory, although these cannot be ruled out. In the majority of cases, the miscalled organism was proximal in the list of organisms tested, further suggesting that the cause was cross‐over contamination between wells when preparing the DNA libraries for sequencing. Such sources of error are of concern in clinical tests, however, in all cases the contaminating organisms represented less than 2% of reads analysed. Taken together, our results suggest that for sequencing technologies currently limited to approximately 500 base pairs, the V1–V2 region can accurately identify a broad selection of clinically important bacteria. In addition, while high fidelity polymerases, careful attention to cross‐contamination and data processing choices are important, the choice of algorithm and reference database appears to have a greater effect on results along with the inherent sequence diversity, or lack thereof, in certain taxonomic orders themselves.

A limitation of our analysis is that results were compared with the identification provided by ATCC without any sequencing confirmation from the ATCC, creating the possibility that the lack of genetic sequence confirmation may be responsible for some of the discrepancies in our identifications (e.g. the *K. kristinae* strain identified as *Kocuria rosae*). Furthermore, we utilized pure strains of bacteria for our study, which would not be possible in a culture‐less approach; however, we made this choice in order to analyse the accuracy and errors that arise with a pure starting material and are thus inherent to the approach.

Low‐density organisms in a mixture are a concern when identifying causes of infection, thus we tested the sensitivity of 16S rRNA gene sequencing through a mixing strategy wherein five pairs of organisms were mixed over a six log range. The pairs represented a hierarchy of four levels of biological difference starting with intra‐species differences and expanded to Gram‐negative versus Gram‐positive species as follows: (i) Same species: MRSA versus MSSA, (ii) different, but difficult to discriminate: *E. coli* vs *S. flexneri*, (iii) Gram‐positive versus Gram‐positive: *S. saprophyticus* versus *S. pyogenes*, and (iv) Gram‐positive vs Gram‐negative: *S. saprophyticus* versus *E. coli*. Of the five mixtures, three generated results indicating the sensitivity for 16S rRNA sequencing is below 0·1% in a binary mixture. However, 16S rRNA gene sequencing could not separate strains of the same species (MSSA versus MRSA), nor *E. coli* from *S. flexneri* with high fidelity. These results were not unexpected, but the mixtures were included to demonstrate and highlight the limitations of the method and allow for future comparison to other methods of identification. With the increasing prevalence of drug‐resistant bacterial strains such as MRSA in clinical infections, the utility of 16S rRNA gene sequencing will increasingly suffer without additional efforts to identify drug resistance (such as inclusion of primers to detect the presence of drug resistance genes). Nonetheless, a 16S rRNA sequencing result showing the presence of *S. aureus* can be useful in that it indicates the isolate should be tested for antibiotic sensitivity. For the three mixtures of organisms that can be reliably identified by 16S rRNA gene sequencing, the results indicate that the method is sensitive to 0·1% with good representation of actual ratios in the mixture. These results could be partially affected by the varying copy number of the 16S rRNA gene between the species mixed, for which we did not compensate; however, the species utilized all have between five and seven 16S rRNA copies per genome, making any skewing due to copy number variation limited in scope.

The accuracy and sensitivity of the V1–V2 hypervariable region on a benchtop sequencer such as the Ion torrent PGM indicate that it may have a place in the clinical setting. However, the misidentifications combined with the lack of drug resistance detection lead us to conclude that while performance was good for 40 of 41 strains, it is not an ideal alternative to culture‐based identification of bacteria. Despite the drawbacks, it is possible that the effort would be worthwhile for a variety of fastidious organisms or emerging microbes. However, given the small size of most bacterial genomes compared to the throughput of the Ion Torrent PGM, it could be feasible to perform whole‐genome sequencing of multiple samples in a single run. Whole‐genome sequencing offers the opportunity to differentiate even closely related strains as well as detect drug resistance as demonstrated by recent studies (Allard *et al*. 2012; Zankari *et al*. 2012; Katz *et al*. 2013; Underwood *et al*. 2013; Joensen *et al*. 2014; Holmes *et al*. 2015; Liu *et al*. 2016; Zhao *et al*. 2016). Given the greater sensitivity and discriminatory power of whole‐genome sequencing, as a next step, we will explore the accuracy and sensitivity of this approach in clinical samples with the eventual goal of culture‐free identification.

## Conflict of Interest

Dr. George S. Watts, Dr. Bonnie L. Hurwitz and Dr. Marvin J. Slepian have a disclosed financial interest in Biomyxx that had no involvement in the work reported here. The terms of this arrangement have been properly disclosed to the University of Arizona and reviewed by the Institutional Review Committee in accordance with its conflict of interest policies.

## Supporting information


**Table S1.** Taxonomic level reached, percent of reads matching most prevalent organism, and identities of misidentified species resulting from sequencing 16S rRNA hypervariable regions V1, V3, V6 and V1–V2 in 41 strain isolates. Tax. Level, taxonomic level reached based on identity of most prevalent organism matched, compared to known identity. % reads in tax. level, percent of total reads represented by the most prevalent species identified. The most prevalent organism is listed if different from the correct organism. S, species; G, genus; F, family; O, order; C, class; K, kingdom; ND, no result due to too few reads
**Table S2.** Taxonomic level reached, percent of reads in consensus call, and consensus identity resulting from sequencing 16S rRNA hypervariable regions V2, V3, V4, V6–7, V8 and V9 generated with the Ion Torrent Metagenomics kit in 11 strain isolates. Sequence data analysis was performed utilizing the Ion Torrent Metagenomics workflow on the Ion Reporter website. S, species; G, genus, F, family, O, order, ND, no result due to lack of mapped reads. Consensus, the percent of reads that mapped to the correct genus for each strain analysed using results from all available hypervariable regions, along with the percent of reads falling into the consensus call
**Table S3.** Taxonomic level and error rate for three data analysis methods: the Ribosomal Database Project SequenceMatch, NCBI blastn, and Ion Torrent's Ion Reporter (IR) Metagenomics analysis workflow for each of 41 strains analysed. Error rate is the percent of reads not matching the most prevalent species identified in each sample. S, species; G, genus; F, family; NA, not applicable. For genus‐ and family‐level calls made by the Metagenomics workflow, the other species or genera detected are listed, respectively
**Table S4.** Full results of blastn and RDP SequenceMatch for each of the 41 strains sequenced. For each analysis method, the number of reads matching a particular organism is listed in descending order for each sample. Total reads that passed matching parameters for each algorithm is shown at the bottom of each list.
