# Supplementary material for: 16S rRNA gene sequencing on a benchtop sequencer: accuracy for identification of clinically important bacteria
Source: J Appl Microbiol. 2017 Nov 7;123(6):1584–96. doi: 10.1111/jam.13590 (PMC5765505; doi:10.1111/jam.13590)
Supplement: Supplementary file 1 — Table S1. Taxonomic level reached, percent of reads matching most prevalent organism, and identities of misidentified species resulting from sequencing 16S rRNA hypervariable regions V1, V3, V6 and V1–V2 in 41 strain isolates. Tax. Level, taxonomic level reached based on identity of most prevalent organism matched, compared to known identity. % reads in tax. level, percent of total reads represented by the most prevalent species identified. The most prevalent organism is listed if different from the correct organism. S, species; G, genus; F, family; O, order; C, class; K, kingdom; ND, no result due to too few reads Table S2. Taxonomic level reached, percent of reads in consensus call, and consensus identity resulting from sequencing 16S rRNA hypervariable regions V2, V3, V4, V6–7, V8 and V9 generated with the Ion Torrent Metagenomics kit in 11 strain isolates. Sequence data analysis was performed utilizing the Ion Torrent Metagenomics workflow on the Ion Reporter website. S, species; G, genus, F, family, O, order, ND, no result due to lack of mapped reads. Consensus, the percent of reads that mapped to the correct genus for each strain analysed using results from all available hypervariable regions, along with the percent of reads falling into the consensus call Table S3. Taxonomic level and error rate for three data analysis methods: the Ribosomal Database Project SequenceMatch, NCBI blastn, and Ion Torrent's Ion Reporter (IR) Metagenomics analysis workflow for each of 41 strains analysed. Error rate is the percent of reads not matching the most prevalent species identified in each sample. S, species; G, genus; F, family; NA, not applicable. For genus‐ and family‐level calls made by the Metagenomics workflow, the other species or genera detected are listed, respectively Table S4. Full results of blastn and RDP SequenceMatch for each of the 41 strains sequenced. For each analysis method, the number of reads matching a particular organism is listed in descending orde [file JAM-123-1584-s001.pdf]

Table S1. Taxonomic level reached, percent of reads matching most prevalent organism, and identities of misidentified species resulting from sequencing 16S rRNA hypervariable regions V1, V3, V6, and V1-V2 in 41 strain isolates. Tax. Level, taxonomic level reached based on identity of most prevalent organism matched, compared to known identity. % reads in tax. level, percent of total reads represented by the most prevalent species identified. The most prevalent organism is listed if different from the correct organism. S, species; G, genus; F, family; O, order; C, class; K, kingdom; ND, no result due to too few reads.

Table S2. Taxonomic level reached, percent of reads in consensus call, and consensus identity resulting from sequencing 16S rRNA hypervariable regions V2, V3, V4, V6-7, V8, and V9 generated with the Ion Torrent Metagenomics kit in 11 strain isolates. Sequence data analysis was performed utilizing the Ion Torrent Metagenomics workflow on the Ion Reporter website. S, species; G, genus, F, family, O, order, ND, no result due to lack of mapped reads. Consensus, the percent of reads that mapped to the correct genus for each strain analyzed using results from all available hypervariable regions, along with the percent of reads falling into the consensus call.

Table S3. Taxonomic level and error rate for three data analysis methods: the Ribosomal Database Project SequenceMatch, NCBI blastn, and Ion Torrent's Ion Reporter (IR) Metagenomics analysis workflow for each of 41 strains analyzed. Error rate is the percent of reads not matching the most prevalent species identified in each sample. S, species; G, genus; F, family; NA, not applicable. For genus and family level calls made by the Metagenomics workflow, the other species or genera detected are listed, respectively.

Table S4. Full results of blastn and RDP SequenceMatch for each of the 41 strains sequenced. For each analysis method, the number of reads matching a particular organism are listed in descending order for each sample. Total reads that passed matching parameters for each algorithm is shown at the bottom of each list.

Organism	V1			V3			
	tax. level	% reads in tax. level	most prevalent organism	tax. level	% reads in tax. level	most prevalent organism	tax. level
Proteus mirabilis **Hauser ATCC 12453**	S	97.2	-	S	99.2	-	K
Staphylococcus aureus **BAA 1721**	S	99.9	-	S	99.5	-	S
Staphylococcus aureus **BAA 1720**	S	99.7	-	S	99.0	-	S
Staphylococcus saprophyticus **ATCC 15305**	G	99.6	*Staphylococcus arlettae*	G	97.8	*Staphylococcus arlettae*	C
Staphylococcus epidermidis **ATCC 35984**	O	99.1	*Geobacillus stearothermophilus*	O	99.1	*Geobacillus stearothermophilus*	O
Enterococcus faecalis **ATCC 51299**	S	98.7	-	F	98.9	*Lactobacillus plantarum*	C
Enterococcus faecalis **ATCC 29212**	S	99.5	-	F	99.5	*Lactobacillus plantarum*	C
Staphylococcus epidermidis **ATCC 12228**	O	99.0	*Geobacillus stearothermophilus*	O	100	*Geobacillus stearothermophilus*	O
Staphylococcus aureus **ATCC 43300**	S	99.7	-	S	99.2	-	S
Enterococcus faecium **ATCC 51559**	G	97.5	*Enterococcus hirae*	F	100	*Lactobacillus fermentum*	K
Streptococcus pneumoniae **ATCC 6305**	G	99.6	*Streptococcus mitis*	G	100	*Streptococcus mitis*	G
Kocuria kristinae **BAA 752**	S	96.9	-	F	95.4	*Arthrobacter crystallopoietes*	S
Escherichia coli **ATCC 25922**	F	4.0	*Enterobacter cloacae*	S	95.3	-	F
Streptococcus pyogenes **ATCC 19615**	S	100	-	S	100	-	S
Streptococcus salivarius **ATCC 19258**	G	98.8	*Streptococcus sanguinis*	O	100	*Enterococcus faecium*	G
Streptococcus equi **ATCC 43079**	S	92.9	-	S	99.4	-	S
Enterococcus saccharolyticus **ATCC 43076**	S	99.8		G	99.9	*Enterococcus sulfureus*	C
Enterococcus casseliflavus **ATCC 700327**	G	99.8	*Enterococcus gallinarum*	G	89.5	*Enterococcus faecalis*	G
Staphylococcus saprophyticus **BAA 750**	G	98.3	*Staphylococcus arlettae*	G	99.2	*Staphylococcus xylosus*	C
Listeria monocytogenes **BAA 751**	G	99.7	*Listeria seeligeri*	G	99.7	*Listeria ivanovii*	G
Streptococcus pneumoniae **ATCC 49619**	G	99.9	*Streptococcus mitis*	G	99.8	*Streptococcus mitis*	G
Staphylococcus sciuri **ATCC 29061**	S	95.1	-	S	99.2	-	G
Klebsiella oxytoca **ATCC 700324**	F	99.1	*Enterobacter ludwigii*	S	55.6	-	C
Stenotrophomonas maltophilia **ATCC 17666**	S	96.9	-	S	98.9	-	F
Elizabethkingia meningoseptica **ATCC 13253**	F	100	*Chryseobacterium elymi*	S	99.3	-	F
Corynebacterium striatum **BAA 1293**	S	99.5	-	S	98.9	-	S
Paenibacillus polymyxa **ATCC 7070**	S	96.7	-	S	95.2	-	S
Oligella ureolytica **ATCC 43534**	S	100	-	S	99.3	-	S
Pseudomonas aeruginosa **ATCC 27853**	S	55.1	-	G	99.6	*Pseudomonas stutzeri*	G
Shigella sonnei **ATCC 25931**	F	99.5	*Enterobacteriaceae bacterium* 458	F	97.1	*Escherichia coli*	F
Ochrobactrum anthropi **BAA 749**	ND	ND	ND	O	95.5	*Ensifer kummerowiae*	G
Listeria monocytogenes **BAA 751**	G	99.2	*Listeria seeligeri*	G	99.7	*Listeria ivanovii*	G
Proteus vulgaris **ATCC 6380**	G	92.1	*Proteus mirabilis*	S	95.7	-	K
Bacteroides ovatus **ATCC 1296**	S	97.8	-	S	98.9	-	S
Clostridium sordellii **ATCC 9714**	ND	ND	ND	O	100	*Romboutsia lituseburensis*	C

Table_S1

Organism	16S rRNA Hypervariable region						
	V2	V3	V4	V6-7	V8	V9	Consensus
Ochrobactrum anthropi	F	ND	F	F	F	ND	47% genus *Ochrobactrum*
Clostridium sordellii	S	G	F	F	F	ND	50% genus *Clostridium*
Enterococcus faecalis	F	F	F	F	F	F	53% genus *Enterococcus*
Streptococcus pneumoniae	G	O	F	F	F	G	56% genus *Streptococcus*
Paenibacillus polymyxa	F	F	F	F	F	ND	62% genus *Paenibacillus*
Streptococcus thermophilus	F	F	F	F	F	G	66% genus *Streptococcus*
Eikenella corrodens	G	F	F	F	F	ND	79% genus *Eikenella*
Enterococcus faecium	F	F	F	F	F	G	81% genus *Enterococcus*
Corynebacterium striatum	G	G	G	G	G	ND	88% genus *Corynebacterium*
Staphylococcus epidermidis	G	G	G	G	G	ND	94% genus *Staphylococcus*
Staphylococcus aureus	S	G	G	G	G	ND	94% genus *Staphylococcus*

Organism	RDP SequenceMatch		NCBI blastn		Ion Reporter Metagenomics workflow	
	tax. level	Error Rate (%)	tax. level	Error Rate (%)	tax. Level	Error Rate (%)
Proteus mirabilis Hauser	S	6.5	S	0.1	S	0.0
Staphylococcus aureus	S	0.9	S	0.8	S	0.0
Staphylococcus aureus	S	1.7	S	1.5	S	0.3
Staphylococcus saprophyticus	S	2.8	S	1.9	S	0.1
Staphylococcus epidermidis	S	0.5	S	1.4	S	0.2
Enterococcus faecalis	S	0.8	S	1.5	S	0.0
Enterococcus faecalis	S	1.1	S	1.4	S	0.1
Staphylococcus epidermidis	S	2.0	S	1.4	S	0.2
Staphylococcus aureus	S	0.1	S	0.8	S	0.0
Enterococcus faecium	S	3.4	G	5.1	G	0.6
Streptococcus pneumoniae	S	0.5	S	1.2	G	0.0
Kocuria kristinae	G	0.3	S	3.8	S	1.8
Escherichia coli	S	1.7	F	39.0	F	0.0
Streptococcus pyogenes	S	0.2	S	0.5	F	0.0
Streptococcus thermophilus	S	1.2	S	0.6	G	0.0
Streptococcus equi	S	2.6	S	2.4	S	0.6
Enterococcus saccharolyticus	S	0.3	S	1.2	S	0.0
Enterococcus casseliflavus	S	38.6	S	1.0	G	0.0
Staphylococcus saprophyticus	S	1.8	S	0.1	S	0.0
Listeria monocytogenes	S	0.8	S	14.5	S	0.0
Streptococcus pneumoniae	S	0.3	S	1.1	G	0.0
Staphylococcus sciuri	S	0.4	S	0.3	S	0.0
Klebsiella oxytoca	S	4.5	S	0.3	S	0.0
Stenotrophomonas maltophilia	S	0.5	S	0.1	G	0.0
Elizabethkingia meningoseptica	S	0.0	S	0.3	S	0.0
Corynebacterium striatum	S	0.1	G	1.0	S	0.0
Paenibacillus polymyxa	S	4.5	S	27.6	G	0.0
Oligella ureolytica	S	0.2	S	0.2	S	0.0
Pseudomonas aeruginosa	S	0.6	S	0.1	S	0.0
Shigella sonnei	S	60.5	F	33.6	F	0.0
Ochrobactrum anthropi	S	4.6	S	0.3	G	0.0
Listeria monocytogenes	S	0.7	S	13.5	S	0.1
Proteus vulgaris	S	2.7	S	12.0	G	0.0
Bacteroides ovatus	S	0.7	S	0.8	S	0.0
Clostridium sordellii	S	0.3	S	0.5	S	0.0
Eikenella corrodens	S	0.2	S	0.0	S	0.0
Clostridium perfringens	S	4.1	S	1.7	S	0.0
Clostridium septicum	S	0.6	S	1.9	S	0.0
Aggregatibacter aphrophilus	S	0.2	S	0.2	S	0.0
Haemophilus influenzae	S	0.4	S	49.3	S	0.0
Neisseria gonorrhoeae	S	0.2	S	0.1	S	0.0

Organism sequenced: *Proteus mirabilis* ATCC 12453			
blastn number of reads matched	blastn organism matched	SequenceMatch number of reads matched	SequenceMatch organism matched
282836	*Proteus mirabilis*	301329	*Proteus mirabilis*
49	*Staphylococcus epidermidis*	52	*Staphylococcus epidermidis*
9	*Staphylococcus aureus*	12	*Proteus vulgaris*
9	*Klebsiella oxytoca*	11	*Klebsiella oxytoca*
8	*Shigella sonnei*	9	*Staphylococcus aureus*
8	*Proteus vulgaris*	9	*Ochrobactrum anthropi*
8	*Ochrobactrum anthropi*	8	*Neisseria gonorrhoeae*
7	*Neisseria gonorrhoeae*	8	*Escherichia coli*
6	*Streptococcus pneumoniae*	7	*Aggregatibacter aphrophilus*
6	*Aggregatibacter aphrophilus*	6	*Streptococcus pneumoniae*
5	*Listeria monocytogenes*	6	*Listeria monocytogenes*
4	*Staphylococcus sciuri*	5	*Staphylococcus sciuri*
4	*Pseudomonas aeruginosa*	5	*Paenibacillus polymyxa*
4	*Proteus hauseri*	4	*Streptococcus pyogenes*
4	*Paenibacillus polymyxa*	4	*Pseudomonas aeruginosa*
3	*Streptococcus pyogenes*	3	*Stenotrophomonas maltophilia*
3	*Stenotrophomonas maltophilia*	3	*Staphylococcus saprophyticus*
3	*Staphylococcus saprophyticus*	3	*Oligella ureolytica*
3	*Shigella dysenteriae*	3	*Enterococcus faecalis*
3	*Oligella ureolytica*	3	*Enterobacteriaceae bacterium*
3	*Enterococcus faecalis*	3	*Elizabethkingia meningoseptica*
2	*Streptococcus equi*	3	*Eikenella corrodens*
2	*Escherichia fergusonii*	2	*Streptococcus salivarius*
2	*Enterococcus saccharolyticus*	2	*Streptococcus equi*
2	*Enterococcus faecium*	2	*Shigella sonnei*
2	*Elizabethkingia meningoseptica*	2	*Shigella flexneri*
2	*Eikenella corrodens*	2	*Kocuria rosea*
1	*Streptococcus thermophilus*	2	*Haemophilus influenzae*
1	*Streptococcus dysgalactiae*	2	*Enterococcus saccharolyticus*
1	*Shigella flexneri*	2	*Enterococcus faecium*
1	*Shigella boydii*	2	*Enterobacter hormaechei*
1	*Enterococcus casseliflavus*	2	*Corynebacterium striatum*
1	*Corynebacterium simulans*	2	*Clostridium septicum*
1	*Clostridium septicum*	1	*Shigella boydii*
283004	Total Reads	1	*Ochrobactrum cytisi*
		1	*Enterococcus casseliflavus*
		1	*Enterobacter ludwigii*
		1	*[Clostridium] sordellii*
		1	*Bacteroides ovatus*
		301524	Total Reads
Organism sequenced: *Staphylococcus aureus* ATCC BAA1721			

blastn number of reads matched	blastn organism matched	SequenceMatch number of reads matched	SequenceMatch organism matched
16496	*Staphylococcus aureus*	17412	*Staphylococcus aureus*
10	*Shigella sonnei*	15	*Listeria monocytogenes*
9	*Listeria monocytogenes*	9	*Streptococcus pneumoniae*
8	*Streptococcus pneumoniae*	9	*Pseudomonas aeruginosa*
8	*Pseudomonas aeruginosa*	9	*Enterococcus saccharolyticus*
8	*Enterococcus saccharolyticus*	8	*Enterococcus faecalis*
8	*Enterococcus faecalis*	8	*[Clostridium] sordellii*
7	*Staphylococcus epidermidis*	7	*Streptococcus pyogenes*
6	*Streptococcus pyogenes*	7	*Staphylococcus sciuri*
6	*Staphylococcus sciuri*	7	*Staphylococcus epidermidis*
6	*Proteus mirabilis*	6	*Proteus mirabilis*
6	*Klebsiella oxytoca*	6	*Klebsiella oxytoca*
4	*Enterococcus casseliflavus*	5	*Enterobacteriaceae bacterium*
4	*Bacteroides ovatus*	5	*Bacteroides ovatus*
3	*Streptococcus equi*	4	*Proteus vulgaris*
3	*Proteus vulgaris*	4	*Paenibacillus polymyxa*
3	*Oligella ureolytica*	4	*Elizabethkingia meningoseptica*
3	*Ochrobactrum anthropi*	3	*Streptococcus equi*
3	*Elizabethkingia meningoseptica*	3	*Shigella sonnei*
3	*[Clostridium] sordellii*	3	*Oligella ureolytica*
2	*Shigella dysenteriae*	3	*Neisseria gonorrhoeae*
2	*Paenibacillus polymyxa*	2	*Shigella flexneri*
2	*Neisseria gonorrhoeae*	2	*Ochrobactrum anthropi*
2	*Enterococcus faecium*	2	*Escherichia coli*
2	*Enterococcus camelliae*	2	*Enterococcus gallinarum*
1	*Streptococcus thermophilus*	2	*Enterococcus faecium*
1	*Escherichia fergusonii*	2	*Enterococcus casseliflavus*
1	*Eikenella corrodens*	2	*Enterobacter hormaechei*
1	*Corynebacterium simulans*	2	*Corynebacterium striatum*
1	*Clostridium septicum*	2	*Clostridium septicum*
1	*Clostridium perfringens*	1	*Streptococcus salivarius*
1	*Bacillus siralis*	1	*Staphylococcus equorum*
16621	Total Reads	1	*Ochrobactrum cytisi*
		1	*Haemophilus influenzae*
		1	*Eikenella corrodens*
		1	*Clostridium perfringens*
		1	*Citrobacter freundii*
		1	*Aggregatibacter aphrophilus*
		17563	Total Reads

Organism sequenced: *Staphylococcus aureus* ATCC BAA1720

blastn number of reads matched	blastn organism matched	SequenceMatch number of reads matched	SequenceMatch organism matched
30799	*Staphylococcus aureus*	32401	*Staphylococcus aureus*
152	*Proteus mirabilis*	162	*Proteus mirabilis*
30	*Klebsiella oxytoca*	44	*Kocuria rosea*
25	*Listeria monocytogenes*	36	*Listeria monocytogenes*
25	*Kocuria kristinae*	30	*Klebsiella oxytoca*
19	*Staphylococcus epidermidis*	20	*Enterococcus saccharolyticus*
18	*Enterococcus saccharolyticus*	19	*Staphylococcus epidermidis*
17	*Streptococcus pyogenes*	18	*Streptococcus pyogenes*
16	*Enterococcus faecalis*	18	*Paenibacillus polymyxa*
13	*Shigella sonnei*	17	*Enterococcus faecalis*
11	*Staphylococcus sciuri*	16	*[Clostridium] sordellii*
10	*Paenibacillus polymyxa*	13	*Bacteroides ovatus*
10	*Neisseria gonorrhoeae*	12	*Staphylococcus sciuri*
10	*Bacteroides ovatus*	11	*Streptococcus pneumoniae*
9	*Streptococcus thermophilus*	10	*Pseudomonas aeruginosa*
8	*Streptococcus pneumoniae*	10	*Neisseria gonorrhoeae*
8	*Pseudomonas aeruginosa*	10	*Clostridium perfringens*
8	*Enterococcus faecium*	9	*Streptococcus salivarius*
8	*Clostridium perfringens*	9	*Streptococcus equi*
7	*[Clostridium] sordellii*	9	*Enterococcus faecium*
6	*Streptococcus equi*	7	*Shigella sonnei*
6	*Ochrobactrum anthropi*	7	*Proteus vulgaris*
6	*Eikenella corrodens*	7	*Ochrobactrum anthropi*
5	*Proteus vulgaris*	7	*Enterobacteriaceae bacterium*
5	*Paenibacillus jamilae*	6	*Eikenella corrodens*
5	*Enterococcus casseliflavus*	5	*Clostridium septicum*
4	*Shigella boydii*	4	*Shigella boydii*
4	*Clostridium septicum*	4	*Elizabethkingia meningoseptica*
3	*Staphylococcus saprophyticus*	3	*Stenotrophomonas maltophilia*
3	*Shigella flexneri*	3	*Staphylococcus saprophyticus*
3	*Elizabethkingia meningoseptica*	3	*Oligella ureolytica*
2	*Stenotrophomonas maltophilia*	3	*Kocuria kristinae*
2	*Proteus hauseri*	3	*Haemophilus influenzae*
2	*Oligella ureolytica*	3	*Enterococcus casseliflavus*
2	*Haemophilus aegyptius*	3	*Corynebacterium striatum*
2	*Enterococcus camelliae*	3	*Citrobacter freundii*
2	*Corynebacterium simulans*	2	*Shigella flexneri*
2	*Aggregatibacter aphrophilus*	2	*Escherichia coli*
1	*Shigella dysenteriae*	2	*Aggregatibacter aphrophilus*
1	*Anoxybacillus voinovskiensis*	1	*Ochrobactrum tritici*
31269	Total Reads	1	*Ochrobactrum cytisi*
		1	*Enterococcus gallinarum*

		1	*Enterobacter ludwigii*
		1	*Enterobacter hormaechei*
		32956	Total Reads
Organism sequenced: *Staphylococcus saprophyticus* ATCC 15305			
blastn number of reads matched	blastn organism matched	SequenceMatch number of reads matched	SequenceMatch organism matched
16149	*Staphylococcus saprophyticus*	16272	*Staphylococcus saprophyticus*
59	*Staphylococcus epidermidis*	95	*Staphylococcus xylosus*
53	*Enterococcus faecalis*	66	*Staphylococcus epidermidis*
31	*Streptococcus pyogenes*	56	*Enterococcus faecalis*
25	*Streptococcus equi*	37	*Streptococcus pyogenes*
14	*Staphylococcus aureus*	30	*Streptococcus equi*
11	*Listeria monocytogenes*	22	*Listeria monocytogenes*
11	*Enterococcus saccharolyticus*	16	*Staphylococcus aureus*
9	*Neisseria gonorrhoeae*	12	*Enterococcus saccharolyticus*
8	*Klebsiella oxytoca*	11	*Neisseria gonorrhoeae*
7	*Staphylococcus sciuri*	9	*Staphylococcus sciuri*
6	*Streptococcus pneumoniae*	9	*Paenibacillus polymyxa*
6	*Pseudomonas aeruginosa*	8	*[Clostridium] sordellii*
5	*Stenotrophomonas maltophilia*	8	*Bacteroides ovatus*
5	*Shigella sonnei*	7	*Streptococcus pneumoniae*
5	*Proteus mirabilis*	7	*Pseudomonas aeruginosa*
4	*Enterococcus faecium*	7	*Klebsiella oxytoca*
4	*Eikenella corrodens*	6	*Proteus mirabilis*
4	*Aggregatibacter aphrophilus*	5	*Stenotrophomonas maltophilia*
3	*Paenibacillus polymyxa*	5	*Ochrobactrum anthropi*
3	*Paenibacillus jamilae*	5	*Enterococcus faecium*
3	*Haemophilus influenzae*	4	*Kocuria rosea*
3	*Clostridium septicum*	4	*Haemophilus influenzae*
3	*Bacteroides ovatus*	4	*Escherichia coli*
2	*Streptococcus thermophilus*	4	*Eikenella corrodens*
2	*Proteus vulgaris*	4	*Aggregatibacter aphrophilus*
2	*Ochrobactrum anthropi*	3	*Shigella sonnei*
2	*Kocuria kristinae*	3	*Proteus vulgaris*
2	*Escherichia fergusonii*	3	*Enterobacteriaceae bacterium*
2	*Enterococcus casseliflavus*	3	*Clostridium septicum*
2	*Corynebacterium simulans*	3	*Citrobacter freundii*
2	*[Clostridium] sordellii*	2	*Streptococcus salivarius*
1	*Streptococcus lutetiensis*	2	*Staphylococcus cohnii*
1	*Staphylococcus capitis*	2	*Shigella flexneri*
1	*Shigella dysenteriae*	2	*Enterococcus casseliflavus*
1	*Shigella boydii*	2	*Corynebacterium striatum*
1	*Oligella ureolytica*	1	*Streptococcus equinus*
1	*Geobacillus stearothermophilus*	1	*Staphylococcus capitis*
1	*Enterococcus camelliae*	1	*Oligella ureolytica*

1	*Elizabethkingia meningoseptica*	1	*Geobacillus stearothermophilus*
1	*Clostridium perfringens*	1	*Elizabethkingia meningoseptica*
1	*Clostridium lundense*	1	*Clostridium perfringens*
1	*Citrobacter koseri*	16744	Total Reads
16458	Total Reads		

Organism sequenced: *Staphylococcus epidermidis* ATCC 35984			
blastn number of reads matched	blastn organism matched	SequenceMatch number of reads matched	SequenceMatch organism matched
19372	*Staphylococcus epidermidis*	20822	*Staphylococcus epidermidis*
138	*Enterococcus faecalis*	151	*Enterococcus faecalis*
12	*Proteus mirabilis*	117	*Staphylococcus aureus*
9	*Streptococcus pyogenes*	12	*Proteus mirabilis*
9	*Staphylococcus aureus*	9	*Streptococcus pyogenes*
9	*Klebsiella oxytoca*	9	*Klebsiella oxytoca*
7	*Staphylococcus sciuri*	8	*Neisseria gonorrhoeae*
7	*Shigella sonnei*	7	*Staphylococcus sciuri*
6	*Neisseria gonorrhoeae*	7	*Ochrobactrum anthropi*
5	*Streptococcus equi*	7	*Listeria monocytogenes*
5	*Staphylococcus saprophyticus*	6	*Escherichia coli*
5	*Ochrobactrum anthropi*	6	*Enterococcus saccharolyticus*
5	*Enterococcus casseliflavus*	5	*Streptococcus pneumoniae*
4	*Listeria monocytogenes*	5	*Streptococcus equi*
4	*Enterococcus saccharolyticus*	5	*Staphylococcus saprophyticus*
3	*Streptococcus pneumoniae*	5	*Haemophilus influenzae*
3	*Stenotrophomonas maltophilia*	5	*Geobacillus stearothermophilus*
3	*Shigella flexneri*	4	*Staphylococcaceae bacterium*
3	*Paenibacillus polymyxa*	4	*Shigella sonnei*
3	*Haemophilus aegyptius*	4	*Paenibacillus polymyxa*
3	*Clostridium perfringens*	4	*Enterococcus faecium*
2	*Streptococcus thermophilus*	4	*Enterococcus casseliflavus*
2	*Shigella dysenteriae*	4	*[Clostridium] sordellii*
2	*Gracilibacillus dipsosauri*	4	*Bacteroides ovatus*
2	*Enterococcus camelliae*	3	*Stenotrophomonas maltophilia*
2	*[Clostridium] sordellii*	3	*Clostridium perfringens*
2	*Bacteroides ovatus*	2	*Streptococcus salivarius*
1	*Pseudomonas aeruginosa*	2	*Proteus vulgaris*
1	*Proteus vulgaris*	2	*Enterobacter hormaechei*
1	*Paenibacillus jamilae*	1	*Shigella flexneri*
1	*Oligella ureolytica*	1	*Pseudomonas aeruginosa*
1	*Haemophilus influenzae*	1	*Oligella ureolytica*
1	*Escherichia fergusonii*	1	*Enterobacteriaceae bacterium*
1	*Enterococcus thailandicus*	1	*Eikenella corrodens*
1	*Enterococcus faecium*	1	*Corynebacterium striatum*
1	*Enterococcus durans*	1	*Citrobacter freundii*
1	*Eikenella corrodens*	1	*Aggregatibacter aphrophilus*

1	*Corynebacterium simulans*	21234	Total Reads
1	*Bacillus amyloliquefaciens*		
1	*Aggregatibacter aphrophilus*		
19640	Total Reads		

Organism sequenced: *Enterococcus faecalis ATCC 51299*

blastn number of reads matched	blastn organism matched	SequenceMatch number of reads matched	SequenceMatch organism matched
32111	*Enterococcus faecalis*	36543	*Enterococcus faecalis*
258	*Gracilibacillus dipsosauri*	28	*Listeria monocytogenes*
19	*Shigella sonnei*	19	*Lactobacillus plantarum*
18	*Enterococcus saccharolyticus*	19	*Enterococcus saccharolyticus*
15	*Streptococcus pyogenes*	16	*Klebsiella oxytoca*
14	*Listeria monocytogenes*	15	*Streptococcus pyogenes*
14	*Klebsiella oxytoca*	15	*[Clostridium] sordellii*
10	*[Clostridium] sordellii*	10	*Streptococcus pneumoniae*
9	*Streptococcus pneumoniae*	10	*Shigella sonnei*
9	*Staphylococcus saprophyticus*	9	*Staphylococcus sciuri*
8	*Staphylococcus sciuri*	9	*Staphylococcus saprophyticus*
8	*Ochrobactrum anthropi*	9	*Ochrobactrum anthropi*
8	*Enterococcus casseliflavus*	9	*Enterococcus faecium*
7	*Staphylococcus epidermidis*	8	*Staphylococcus epidermidis*
7	*Oligella ureolytica*	8	*Escherichia coli*
7	*Clostridium septicum*	8	*Bacteroides ovatus*
6	*Proteus mirabilis*	7	*Paenibacillus polymyxa*
6	*Eikenella corrodens*	7	*Oligella ureolytica*
5	*Enterococcus faecium*	7	*Enterococcus casseliflavus*
5	*Bacteroides ovatus*	7	*Enterobacteriaceae bacterium*
5	*Aggregatibacter aphrophilus*	7	*Eikenella corrodens*
4	*Paenibacillus polymyxa*	7	*Clostridium septicum*
4	*Neisseria gonorrhoeae*	6	*Proteus vulgaris*
3	*Streptococcus thermophilus*	6	*Proteus mirabilis*
3	*Streptococcus equi*	6	*Aggregatibacter aphrophilus*
3	*Staphylococcus aureus*	4	*Neisseria gonorrhoeae*
3	*Shigella dysenteriae*	4	*Haemophilus influenzae*
3	*Proteus vulgaris*	4	*Enterococcus gallinarum*
3	*Haemophilus aegyptius*	3	*Streptococcus salivarius*
3	*Gracilibacillus ureilyticus*	3	*Streptococcus equi*
3	*Bacillus vietnamensis*	3	*Staphylococcus aureus*
2	*Stenotrophomonas maltophilia*	3	*Elizabethkingia meningoseptica*
2	*Shigella flexneri*	3	*Corynebacterium striatum*
2	*Elizabethkingia meningoseptica*	2	*Stenotrophomonas maltophilia*
2	*Corynebacterium simulans*	2	*Enterobacter hormaechei*
2	*Citrobacter koseri*	1	*Shigella flexneri*
1	*Shigella boydii*	1	*Shigella boydii*
1	*Pseudomonas aeruginosa*	1	*Rheinheimera texasensis*

1	*Paenibacillus jamilae*	1	*Pseudomonas aeruginosa*
1	*Escherichia fergusonii*	1	*Enterobacter ludwigii*
1	*Enterococcus durans*	1	*Enterobacter cloacae*
32596	Total Reads	1	*Clostridium perfringens*
		36833	Total Reads

Organism sequenced: *Enterococcus faecalis* ATCC 29212			
blastn number of reads matched	blastn organism matched	SequenceMatch number of reads matched	SequenceMatch organism matched
43861	*Enterococcus faecalis*	49009	*Enterococcus faecalis*
174	*Gracilibacillus dipsosauri*	184	*Staphylococcus epidermidis*
165	*Staphylococcus epidermidis*	41	*Listeria monocytogenes*
28	*Streptococcus equi*	29	*Streptococcus equi*
27	*Listeria monocytogenes*	29	*Enterococcus saccharolyticus*
26	*Klebsiella oxytoca*	27	*Klebsiella oxytoca*
26	*Enterococcus saccharolyticus*	26	*Streptococcus pyogenes*
23	*Streptococcus pyogenes*	21	*Lactobacillus plantarum*
9	*Streptococcus pneumoniae*	11	*Streptococcus pneumoniae*
9	*Neisseria gonorrhoeae*	11	*[Clostridium] sordellii*
9	*Enterococcus casseliflavus*	10	*Proteus mirabilis*
8	*Staphylococcus aureus*	10	*Haemophilus influenzae*
8	*Shigella sonnei*	9	*Shigella sonnei*
8	*Proteus mirabilis*	9	*Neisseria gonorrhoeae*
7	*Staphylococcus sciuri*	9	*Enterococcus faecium*
6	*Enterococcus faecium*	8	*Staphylococcus aureus*
6	*Eikenella corrodens*	8	*Corynebacterium striatum*
5	*Corynebacterium simulans*	7	*Staphylococcus sciuri*
5	*Clostridium septicum*	7	*Ochrobactrum anthropi*
5	*Clostridium perfringens*	7	*Enterococcus casseliflavus*
4	*Stenotrophomonas maltophilia*	7	*Eikenella corrodens*
4	*Proteus vulgaris*	6	*Proteus vulgaris*
4	*[Clostridium] sordellii*	6	*Paenibacillus polymyxa*
4	*Bacteroides ovatus*	5	*Clostridium septicum*
4	*Aggregatibacter aphrophilus*	5	*Clostridium perfringens*
3	*Shigella dysenteriae*	5	*Bacteroides ovatus*
3	*Ochrobactrum anthropi*	5	*Aggregatibacter aphrophilus*
3	*Haemophilus influenzae*	4	*Stenotrophomonas maltophilia*
3	*Escherichia fergusonii*	4	*Escherichia coli*
3	*Enterococcus camelliae*	4	*Enterobacteriaceae bacterium*
2	*Streptococcus thermophilus*	3	*Enterobacter hormaechei*
2	*Shigella flexneri*	3	*Elizabethkingia meningoseptica*
2	*Shigella boydii*	2	*Streptococcus salivarius*
2	*Pseudomonas aeruginosa*	2	*Shigella flexneri*
2	*Oligella ureolytica*	2	*Pseudomonas aeruginosa*
2	*Haemophilus aegyptius*	2	*Oligella ureolytica*
1	*Paenibacillus polymyxa*	2	*Enterococcus gallinarum*

1	*Paenibacillus peoriae*	1	*Citrobacter freundii*
1	*Paenibacillus jamilae*	49540	Total Reads
1	*Enterococcus sulfureus*		
1	*Elizabethkingia meningoseptica*		
1	*Citrobacter koseri*		
1	*Bacillus vietnamensis*		
1	*Bacillus megaterium*		
44470	Total Reads		

Organism sequenced: *Staphylococcus epidermidis* ATCC 12228			
blastn number of reads matched	blastn organism matched	SequenceMatch number of reads matched	SequenceMatch organism matched
27732	*Staphylococcus epidermidis*	30029	*Staphylococcus epidermidis*
224	*Streptococcus equi*	239	*Streptococcus equi*
47	*Streptococcus pyogenes*	141	*Staphylococcus aureus*
16	*Listeria monocytogenes*	54	*Streptococcus pyogenes*
12	*Klebsiella oxytoca*	21	*Listeria monocytogenes*
7	*Staphylococcus sciuri*	12	*Klebsiella oxytoca*
6	*Shigella sonnei*	10	*Shigella sonnei*
6	*Clostridium septicum*	9	*Staphylococcus sciuri*
5	*Streptococcus pneumoniae*	9	*Bacteroides ovatus*
5	*Proteus mirabilis*	7	*Streptococcus pneumoniae*
5	*Neisseria gonorrhoeae*	7	*Staphylococcaceae bacterium*
5	*Elizabethkingia meningoseptica*	7	*[Clostridium] sordellii*
4	*Streptococcus thermophilus*	6	*Proteus mirabilis*
4	*Staphylococcus capitis*	6	*Paenibacillus polymyxa*
4	*Staphylococcus aureus*	6	*Ochrobactrum anthropi*
4	*Enterococcus faecium*	6	*Elizabethkingia meningoseptica*
4	*Bacteroides ovatus*	6	*Clostridium septicum*
3	*Stenotrophomonas maltophilia*	5	*Streptococcus salivarius*
3	*Staphylococcus saprophyticus*	5	*Neisseria gonorrhoeae*
3	*Shigella boydii*	5	*Enterococcus saccharolyticus*
3	*Ochrobactrum anthropi*	4	*Haemophilus influenzae*
3	*Enterococcus saccharolyticus*	4	*Enterococcus faecium*
3	*[Clostridium] sordellii*	3	*Stenotrophomonas maltophilia*
2	*Oligella ureolytica*	3	*Staphylococcus saprophyticus*
2	*Enterococcus faecalis*	3	*Enterococcus faecalis*
2	*Enterococcus casseliflavus*	2	*Oligella ureolytica*
2	*Enterococcus camelliae*	2	*Geobacillus stearothermophilus*
2	*Citrobacter koseri*	2	*Enterococcus gallinarum*
1	*Shigella dysenteriae*	2	*Enterococcus casseliflavus*
1	*Pseudomonas aeruginosa*	2	*Eikenella corrodens*
1	*Paenibacillus polymyxa*	2	*Clostridium perfringens*
1	*Paenibacillus jamilae*	1	*Shigella flexneri*
1	*Haemophilus aegyptius*	1	*Pseudomonas aeruginosa*

1	*Enterococcus sulfureus*	1	*Kocuria rosea*
1	*Eikenella corrodens*	1	*Escherichia coli*
1	*Corynebacterium simulans*	1	*Enterobacteriaceae bacterium*
1	*Clostridium perfringens*	1	*Enterobacter hormaechei*
1	*Bacillus composti*	1	*Corynebacterium striatum*
1	*Aggregatibacter aphrophilus*	1	*Citrobacter freundii*
28129	Total Reads	1	*Aggregatibacter aphrophilus*
		30628	Total Reads

Organism sequenced: *Staphylococcus aureus* ATCC 43300

blastn number of reads matched	blastn organism matched	SequenceMatch number of reads matched	SequenceMatch organism matched
32163	*Staphylococcus aureus*	33478	*Staphylococcus aureus*
38	*Klebsiella oxytoca*	40	*Klebsiella oxytoca*
20	*Streptococcus thermophilus*	26	*Listeria monocytogenes*
19	*Enterococcus saccharolyticus*	23	*Enterococcus saccharolyticus*
17	*Listeria monocytogenes*	20	*Streptococcus salivarius*
15	*Streptococcus pneumoniae*	19	*Staphylococcus sciuri*
14	*Staphylococcus sciuri*	18	*Streptococcus pneumoniae*
13	*Shigella sonnei*	17	*[Clostridium] sordellii*
13	*Clostridium septicum*	14	*Paenibacillus polymyxa*
11	*Proteus mirabilis*	13	*Clostridium septicum*
10	*Pseudomonas aeruginosa*	13	*Bacteroides ovatus*
10	*Bacteroides ovatus*	12	*Shigella sonnei*
9	*[Clostridium] sordellii*	12	*Proteus mirabilis*
8	*Staphylococcus epidermidis*	11	*Pseudomonas aeruginosa*
7	*Proteus vulgaris*	11	*Ochrobactrum anthropi*
7	*Ochrobactrum anthropi*	10	*Staphylococcus epidermidis*
6	*Enterococcus casseliflavus*	9	*Proteus vulgaris*
5	*Paenibacillus polymyxa*	7	*Enterococcus faecalis*
5	*Enterococcus faecalis*	7	*Corynebacterium striatum*
4	*Paenibacillus jamilae*	6	*Haemophilus influenzae*
4	*Eikenella corrodens*	6	*Escherichia coli*
3	*Streptococcus pyogenes*	6	*Enterococcus casseliflavus*
3	*Streptococcus equi*	5	*Enterobacteriaceae bacterium*
3	*Stenotrophomonas maltophilia*	5	*Eikenella corrodens*
3	*Shigella dysenteriae*	4	*Streptococcus equi*
3	*Oligella ureolytica*	4	*Stenotrophomonas maltophilia*
3	*Neisseria gonorrhoeae*	3	*Streptococcus pyogenes*
3	*Elizabethkingia meningoseptica*	3	*Oligella ureolytica*
2	*Shigella boydii*	3	*Neisseria gonorrhoeae*
2	*Haemophilus influenzae*	3	*Enterococcus faecium*
2	*Escherichia fergusonii*	3	*Elizabethkingia meningoseptica*
2	*Corynebacterium simulans*	3	*Aggregatibacter aphrophilus*
2	*Aggregatibacter aphrophilus*	2	*Enterobacter hormaechei*

1	*Staphylococcus saprophyticus*	2	*Clostridium perfringens*
1	*Shigella flexneri*	2	*Citrobacter freundii*
1	*Enterococcus faecium*	1	*Staphylococcus saprophyticus*
1	*Enterococcus camelliae*	1	*Enterococcus gallinarum*
1	*Clostridium perfringens*	33822	Total Reads
1	*Clostridium lundense*		
1	*Citrobacter koseri*		
32436	Total Reads		

Organism sequenced: *Enterococcus faecium* ATCC 51559

blastn number of reads matched	blastn organism matched	SequenceMatch number of reads matched	SequenceMatch organism matched
49230	*Enterococcus faecium*	57571	*Enterococcus faecium*
1063	*Enterococcus durans*	821	*Staphylococcus aureus*
614	*Staphylococcus aureus*	420	*Streptococcus salivarius*
409	*Streptococcus thermophilus*	279	*Enterococcus faecalis*
281	*Enterococcus hirae*	209	*Enterococcus hirae*
32	*Enterococcus canis*	52	*Lactobacillus helveticus*
32	*Clostridium lundense*	24	*Enterococcus azikeevi*
25	*Enterococcus thailandicus*	21	*Listeria monocytogenes*
15	*Klebsiella oxytoca*	17	*Enterococcus saccharolyticus*
13	*Shigella sonnei*	15	*Klebsiella oxytoca*
13	*Enterococcus saccharolyticus*	14	*Paenibacillus polymyxa*
11	*Streptococcus pneumoniae*	12	*Streptococcus pneumoniae*
11	*Listeria monocytogenes*	10	*[Clostridium] sordellii*
9	*Paenibacillus polymyxa*	9	*Streptococcus pyogenes*
8	*Staphylococcus epidermidis*	9	*Staphylococcus epidermidis*
8	*Enterococcus faecalis*	9	*Neisseria gonorrhoeae*
7	*Neisseria gonorrhoeae*	8	*Shigella sonnei*
6	*Streptococcus pyogenes*	8	*Corynebacterium striatum*
6	*Proteus mirabilis*	7	*Proteus mirabilis*
6	*Corynebacterium simulans*	7	*Ochrobactrum anthropi*
5	*Streptococcus equi*	6	*Streptococcus equi*
5	*Stenotrophomonas maltophilia*	6	*Pseudomonas aeruginosa*
5	*Staphylococcus sciuri*	5	*Stenotrophomonas maltophilia*
5	*Pseudomonas aeruginosa*	5	*Staphylococcus sciuri*
5	*[Clostridium] sordellii*	5	*Escherichia coli*
5	*Clostridium septicum*	5	*Enterococcus durans*
4	*Proteus vulgaris*	5	*Clostridium septicum*
4	*Enterococcus camelliae*	4	*Proteus vulgaris*
4	*Aggregatibacter aphrophilus*	4	*Haemophilus influenzae*
3	*Staphylococcus saprophyticus*	4	*Enterococcus casseliflavus*
3	*Ochrobactrum anthropi*	4	*Enterococcus canis*
3	*Enterococcus casseliflavus*	4	*Eikenella corrodens*
3	*Eikenella corrodens*	4	*Bacteroides ovatus*
3	*Clostridium perfringens*	4	*Aggregatibacter aphrophilus*

2	*Paenibacillus jamilae*	3	*Staphylococcus saprophyticus*
2	*Oligella ureolytica*	2	*Oligella ureolytica*
2	*Haemophilus influenzae*	2	*Enterobacteriaceae bacterium*
1	*Streptococcus mitis*	2	*Elizabethkingia meningoseptica*
1	*Shigella flexneri*	2	*Clostridium perfringens*
1	*Shigella dysenteriae*	1	*Streptococcus oralis*
1	*Shigella boydii*	1	*Staphylococcus warneri*
1	*Haemophilus aegyptius*	1	*Shigella flexneri*
1	*Escherichia fergusonii*	1	*Shigella boydii*
1	*Elizabethkingia meningoseptica*	1	*Enterococcus lactis*
1	*Bacteroides ovatus*	1	*Clostridiaceae bacterium*
51870	Total Reads	1	*Citrobacter freundii*
		59605	Total Reads

Organism sequenced: *Streptococcus pneumoniae* ATCC 6305

blastn number of reads matched	blastn organism matched	SequenceMatch number of reads matched	SequenceMatch organism matched
55850	*Streptococcus pneumoniae*	64780	*Streptococcus pneumoniae*
399	*Streptococcus mitis*	42	*Streptococcus mitis*
35	*Streptococcus pseudopneumoniae*	41	*Listeria monocytogenes*
26	*Listeria monocytogenes*	21	*Escherichia coli*
20	*Klebsiella oxytoca*	19	*Klebsiella oxytoca*
15	*Shigella sonnei*	15	*Streptococcus salivarius*
13	*Streptococcus thermophilus*	15	*Enterococcus saccharolyticus*
12	*Staphylococcus sciuri*	14	*Staphylococcus sciuri*
12	*Shigella dysenteriae*	11	*Streptococcus pyogenes*
11	*Enterococcus saccharolyticus*	10	*Shigella sonnei*
10	*Streptococcus pyogenes*	10	*Enterococcus faecium*
10	*Enterococcus casseliflavus*	8	*Staphylococcus epidermidis*
8	*Staphylococcus epidermidis*	8	*Proteus mirabilis*
8	*Neisseria gonorrhoeae*	8	*Neisseria gonorrhoeae*
7	*Proteus mirabilis*	7	*Proteus vulgaris*
7	*Enterococcus faecium*	7	*Paenibacillus polymyxa*
6	*Escherichia fergusonii*	7	*Enterococcus casseliflavus*
6	*Enterococcus camelliae*	7	*[Clostridium] sordellii*
5	*[Clostridium] sordellii*	6	*Eikenella corrodens*
4	*Stenotrophomonas maltophilia*	5	*Stenotrophomonas maltophilia*
4	*Shigella flexneri*	5	*Pseudomonas aeruginosa*
4	*Pseudomonas aeruginosa*	5	*Corynebacterium striatum*
4	*Ochrobactrum anthropi*	5	*Clostridium perfringens*
4	*Elizabethkingia meningoseptica*	5	*Bacteroides ovatus*
4	*Corynebacterium simulans*	4	*Streptococcus equi*
4	*Clostridium perfringens*	4	*Ochrobactrum anthropi*
3	*Staphylococcus aureus*	4	*Haemophilus influenzae*
3	*Proteus vulgaris*	4	*Enterobacteriaceae bacterium*
3	*Paenibacillus jamilae*	4	*Enterobacter hormaechei*

3	*Eikenella corrodens*	4	*Elizabethkingia meningoseptica*
3	*Clostridium septicum*	4	*Clostridium septicum*
3	*Aggregatibacter aphrophilus*	4	*Aggregatibacter aphrophilus*
2	*Streptococcus equi*	3	*Streptococcus pseudopneumoniae*
2	*Staphylococcus saprophyticus*	3	*Staphylococcus aureus*
2	*Paenibacillus polymyxa*	3	*Oligella ureolytica*
2	*Oligella ureolytica*	3	*Enterococcus gallinarum*
2	*Kocuria kristinae*	3	*Enterococcus faecalis*
2	*Haemophilus aegyptius*	3	*Citrobacter freundii*
2	*Bacteroides ovatus*	2	*Staphylococcus saprophyticus*
1	*Shigella boydii*	2	*Kocuria rosea*
1	*Haemophilus influenzae*	1	*Staphylococcus warneri*
1	*Enterococcus thailandicus*	1	*Shigella boydii*
1	*Enterococcus faecalis*	1	*Ochrobactrum cytisi*
56524	Total Reads	1	*Enterobacter cloacae*
		65119	Total Reads

Organism sequenced: *Kocuria kristinae* ATCC BAA 752

blastn number of reads matched	blastn organism matched	SequenceMatch number of reads matched	SequenceMatch organism matched
51547	*Kocuria kristinae*	91301	*Kocuria rosea*
1565	*Proteus mirabilis*	1691	*Proteus mirabilis*
270	*Pseudoclavibacter faecalis*	1660	*Kocuria kristinae*
23	*Klebsiella oxytoca*	28	*Klebsiella oxytoca*
19	*Streptococcus pyogenes*	25	*Listeria monocytogenes*
18	*Enterococcus faecalis*	20	*Streptococcus pyogenes*
16	*Streptococcus pneumoniae*	20	*Streptococcus pneumoniae*
14	*Listeria monocytogenes*	18	*Enterococcus faecalis*
12	*Enterococcus faecium*	15	*Enterococcus saccharolyticus*
11	*Shigella sonnei*	12	*Enterococcus faecium*
11	*Enterococcus saccharolyticus*	10	*Staphylococcus aureus*
10	*Staphylococcus aureus*	9	*Corynebacterium striatum*
8	*Corynebacterium simulans*	8	*Streptococcus equi*
7	*Streptococcus equi*	8	*Paenibacillus polymyxa*
5	*Staphylococcus epidermidis*	8	*Ochrobactrum anthropi*
5	*Proteus vulgaris*	8	*Haemophilus influenzae*
4	*Stenotrophomonas maltophilia*	7	*Enterobacteriaceae bacterium*
4	*Staphylococcus sciuri*	6	*Escherichia coli*
4	*Shigella dysenteriae*	6	*[Clostridium] sordellii*
4	*Paenibacillus polymyxa*	5	*Stenotrophomonas maltophilia*
4	*Ochrobactrum anthropi*	5	*Staphylococcus epidermidis*
4	*Neisseria gonorrhoeae*	5	*Shigella sonnei*
3	*Shigella flexneri*	5	*Proteus vulgaris*
3	*Elizabethkingia meningoseptica*	5	*Neisseria gonorrhoeae*
3	*Eikenella corrodens*	4	*Staphylococcus sciuri*
3	*[Clostridium] sordellii*	3	*Pseudomonas aeruginosa*

3	*Bacteroides ovatus*	3	*Enterococcus gallinarum*
3	*Aggregatibacter aphrophilus*	3	*Elizabethkingia meningoseptica*
2	*Streptococcus thermophilus*	3	*Eikenella corrodens*
2	*Pseudomonas aeruginosa*	3	*Bacteroides ovatus*
2	*Paenibacillus jamilae*	3	*Aggregatibacter aphrophilus*
2	*Oligella ureolytica*	2	*Streptococcus salivarius*
2	*Haemophilus influenzae*	2	*Oligella ureolytica*
2	*Haemophilus aegyptius*	2	*Enterococcus casseliflavus*
2	*Enterococcus casseliflavus*	1	*Staphylococcus saprophyticus*
2	*Enterococcus camelliae*	1	*Shigella flexneri*
1	*Staphylococcus saprophyticus*	1	*Enterobacter ludwigii*
1	*Shigella boydii*	1	*Enterobacter hormaechei*
1	*Listeria innocua*	1	*Clostridium septicum*
1	*Gracilibacillus dipsosauri*	1	*Clostridium perfringens*
1	*Enterococcus gallinarum*	1	*Citrobacter freundii*
1	*Clostridium septicum*	94920	Total Reads
1	*Citrobacter koseri*		
1	*Bacillus composti*		
53607	Total Reads		

Organism sequenced: *Escherichia coli* ATCC 25922			
blastn number of reads matched	blastn organism matched	SequenceMatch number of reads matched	SequenceMatch organism matched
28830	*Shigella dysenteriae*	53767	*Escherichia coli*
11592	*Shigella sonnei*	172	*Shigella sonnei*
6003	*Escherichia fergusonii*	133	*Enterobacteriaceae bacterium*
283	*Escherichia coli*	98	*Enterobacter cloacae*
246	*Shigella flexneri*	36	*Klebsiella oxytoca*
56	*Enterobacter cancerogenus*	28	*Escherichia fergusonii*
42	*Citrobacter freundii*	19	*Staphylococcus aureus*
37	*Klebsiella oxytoca*	11	*Proteus mirabilis*
25	*Kosakonia cowanii*	10	*Shigella flexneri*
19	*Staphylococcus aureus*	9	*Enterobacter hormaechei*
11	*Shigella boydii*	8	*Stenotrophomonas maltophilia*
11	*Proteus mirabilis*	8	*Pseudomonas aeruginosa*
7	*Stenotrophomonas maltophilia*	8	*Neisseria gonorrhoeae*
7	*Staphylococcus sciuri*	7	*Staphylococcus sciuri*
7	*Enterobacter mori*	7	*Listeria monocytogenes*
6	*Pseudomonas aeruginosa*	5	*Shigella boydii*
6	*Neisseria gonorrhoeae*	5	*Ochrobactrum anthropi*
5	*Enterococcus saccharolyticus*	5	*Enterococcus saccharolyticus*
4	*Ochrobactrum anthropi*	5	*Enterobacter asburiae*
4	*Listeria monocytogenes*	5	*Eikenella corrodens*
4	*Clostridium septicum*	5	*Aggregatibacter aphrophilus*
4	*Citrobacter koseri*	4	*Paenibacillus polymyxa*

4	*Aggregatibacter aphrophilus*	4	*Clostridium septicum*
3	*Streptococcus pneumoniae*	3	*Streptococcus pneumoniae*
3	*Enterococcus faecalis*	3	*Shigella dysenteriae*
3	*Elizabethkingia meningoseptica*	3	*Proteus vulgaris*
3	*Eikenella corrodens*	3	*Enterococcus faecalis*
2	*Shimwellia blattae*	3	*Elizabethkingia meningoseptica*
2	*Proteus vulgaris*	3	*Bacteroides ovatus*
2	*Paenibacillus jamilae*	2	*Ochrobactrum cytisi*
2	*Haemophilus influenzae*	2	*Haemophilus influenzae*
2	*Enterococcus faecium*	2	*Enterococcus faecium*
2	*[Clostridium] sordellii*	2	*Enterobacter ludwigii*
2	*Bacteroides ovatus*	2	*[Clostridium] sordellii*
1	*Streptococcus thermophilus*	2	*Citrobacter freundii*
1	*Streptococcus pyogenes*	1	*Streptococcus salivarius*
1	*Staphylococcus saprophyticus*	1	*Streptococcus pyogenes*
1	*Paenibacillus polymyxa*	1	*Staphylococcus saprophyticus*
1	*Oligella ureolytica*	1	*Proteus hauseri*
1	*Enterococcus casseliflavus*	1	*Pantoea agglomerans*
1	*Enterococcus camelliae*	1	*Oligella ureolytica*
1	*Enterobacter hormaechei*	1	*Leclercia adecarboxylata*
1	*Dickeya zeae*	1	*Enterococcus casseliflavus*
1	*Corynebacterium simulans*	1	*Enterobacter cancerogenus*
47249	Total Reads	1	*Corynebacterium striatum*
		1	*Citrobacter braakii*
		54400	Total Reads

Organism sequenced: *Streptococcus pyogenes* ATCC 19615

blastn number of reads matched	blastn organism matched	SequenceMatch number of reads matched	SequenceMatch organism matched
96828	*Streptococcus pyogenes*	109958	*Streptococcus pyogenes*
314	*Streptococcus dysgalactiae*	34	*Listeria monocytogenes*
21	*Listeria monocytogenes*	21	*Streptococcus pneumoniae*
13	*Streptococcus pneumoniae*	13	*Enterococcus saccharolyticus*
12	*Klebsiella oxytoca*	12	*Klebsiella oxytoca*
9	*Enterococcus saccharolyticus*	9	*Ochrobactrum anthropi*
8	*Shigella sonnei*	8	*Proteus mirabilis*
7	*Staphylococcus sciuri*	8	*Paenibacillus polymyxa*
6	*Streptococcus thermophilus*	8	*Bacteroides ovatus*
6	*Streptococcus oralis*	7	*Neisseria gonorrhoeae*
6	*Streptococcus mitis*	7	*Haemophilus influenzae*
6	*Pseudomonas aeruginosa*	6	*Staphylococcus sciuri*
6	*Ochrobactrum anthropi*	6	*Staphylococcus aureus*
6	*Neisseria gonorrhoeae*	6	*Pseudomonas aeruginosa*
6	*Enterococcus casseliflavus*	6	*Enterococcus faecium*
6	*Aggregatibacter aphrophilus*	6	*Eikenella corrodens*

5	*Proteus mirabilis*	6	*Clostridium perfringens*
5	*Oligella ureolytica*	6	*Aggregatibacter aphrophilus*
5	*Enterococcus faecalis*	5	*Streptococcus salivarius*
5	*Eikenella corrodens*	5	*Staphylococcus epidermidis*
5	*Clostridium septicum*	5	*Shigella sonnei*
4	*Streptococcus equi*	5	*Oligella ureolytica*
4	*Staphylococcus epidermidis*	5	*Kocuria rosea*
4	*Staphylococcus aureus*	5	*Enterococcus faecalis*
4	*Paenibacillus polymyxa*	5	*[Clostridium] sordellii*
4	*Haemophilus influenzae*	5	*Clostridium septicum*
4	*Enterococcus faecium*	4	*Streptococcus equi*
4	*Enterococcus camelliae*	4	*Enterobacteriaceae bacterium*
4	*Clostridium perfringens*	4	*Enterobacter hormaechei*
4	*Bacteroides ovatus*	3	*Stenotrophomonas maltophilia*
3	*Kocuria kristinae*	3	*Enterococcus gallinarum*
2	*Stenotrophomonas maltophilia*	3	*Enterococcus casseliflavus*
2	*Shigella boydii*	3	*Elizabethkingia meningoseptica*
2	*Paenibacillus jamilae*	2	*Proteus vulgaris*
2	*Escherichia fergusonii*	1	*Staphylococcus stepanovicii*
2	*Elizabethkingia meningoseptica*	1	*Staphylococcus saprophyticus*
1	*Staphylococcus saprophyticus*	1	*Shigella boydii*
1	*Proteus vulgaris*	1	*Paenibacillus jamilae*
1	*Proteus penneri*	1	*Grimontella senegalensis*
1	*Escherichia coli*	1	*Escherichia coli*
1	*Enterobacter cancerogenus*	1	*Corynebacterium striatum*
1	*Corynebacterium simulans*	1	*Citrobacter freundii*
1	*Bartonella clarridgeiae*	110201	Total Reads
1	*Bacillus composti*		
97342	Total Reads		

Organism sequenced: *Streptococcus salivarius* ATCC 19258

blastn number of reads matched	blastn organism matched	SequenceMatch number of reads matched	SequenceMatch organism matched
66779	*Streptococcus thermophilus*	68117	*Streptococcus salivarius*
39	*Listeria monocytogenes*	292	*Enterococcus faecium*
32	*Streptococcus salivarius*	55	*Listeria monocytogenes*
27	*Streptococcus pneumoniae*	39	*Staphylococcus warneri*
26	*Enterococcus saccharolyticus*	34	*Streptococcus pneumoniae*
24	*Klebsiella oxytoca*	31	*Enterococcus saccharolyticus*
19	*Neisseria gonorrhoeae*	25	*Klebsiella oxytoca*
18	*Streptococcus pyogenes*	23	*Streptococcus pyogenes*
17	*Ochrobactrum anthropi*	20	*Neisseria gonorrhoeae*
16	*Staphylococcus aureus*	19	*Ochrobactrum anthropi*
14	*Proteus vulgaris*	18	*Proteus vulgaris*
13	*Shigella sonnei*	17	*Staphylococcus aureus*
13	*Proteus mirabilis*	17	*Paenibacillus polymyxa*

12	*Staphylococcus sciuri*	16	*Shigella sonnei*
11	*Staphylococcus epidermidis*	14	*Staphylococcus sciuri*
11	*Enterococcus casseliflavus*	14	*Staphylococcus epidermidis*
9	*Paenibacillus polymyxa*	14	*Bacteroides ovatus*
8	*Enterococcus faecalis*	13	*Proteus mirabilis*
8	*Bacteroides ovatus*	12	*Corynebacterium striatum*
7	*Pseudomonas aeruginosa*	10	*Pseudomonas aeruginosa*
6	*Eikenella corrodens*	10	*Enterococcus casseliflavus*
6	*Corynebacterium simulans*	9	*Enterococcus faecalis*
5	*Shigella boydii*	8	*Streptococcus equi*
5	*Haemophilus aegyptius*	8	*Haemophilus influenzae*
5	*Enterococcus faecium*	7	*Enterobacteriaceae bacterium*
5	*Enterococcus camelliae*	7	*Eikenella corrodens*
5	*Clostridium septicum*	6	*Stenotrophomonas maltophilia*
4	*Streptococcus equi*	6	*Oligella ureolytica*
4	*Staphylococcus saprophyticus*	6	*Escherichia coli*
4	*Shigella dysenteriae*	6	*Arthrobacter agilis*
4	*Oligella ureolytica*	5	*Staphylococcus saprophyticus*
4	*Clostridium perfringens*	5	*Enterococcus gallinarum*
4	*Aggregatibacter aphrophilus*	5	*Clostridium septicum*
3	*Stenotrophomonas maltophilia*	4	*Elizabethkingia meningoseptica*
3	*Paenibacillus jamilae*	4	*[Clostridium] sordellii*
3	*Citrobacter koseri*	4	*Clostridium perfringens*
3	*Arthrobacter agilis*	4	*Aggregatibacter aphrophilus*
2	*Shigella flexneri*	3	*Kocuria rosea*
2	*Escherichia fergusonii*	2	*Shigella flexneri*
2	*Elizabethkingia meningoseptica*	2	*Proteus hauseri*
1	*Proteus hauseri*	2	*Ochrobactrum cytisi*
1	*Haemophilus influenzae*	2	*Enterobacter hormaechei*
1	*[Clostridium] sordellii*	1	*Paenibacillus jamilae*
1	*Clostridium intestinale*	1	*Citrobacter freundii*
1	*Bacillus vietnamensis*	68917	Total Reads
67187	Total Reads		
Organism sequenced: *Streptococcus equi* ATCC 43079			
blastn number of reads matched	blastn organism matched	SequenceMatch number of reads matched	SequenceMatch organism matched
44007	*Streptococcus equi*	47935	*Streptococcus equi*
473	*Streptococcus pyogenes*	544	*Streptococcus pyogenes*
369	*Staphylococcus capitis*	342	*Staphylococcus epidermidis*
24	*Listeria monocytogenes*	73	*Staphylococcus capitis*
19	*Streptococcus pneumoniae*	31	*Listeria monocytogenes*
19	*Klebsiella oxytoca*	22	*Streptococcus pneumoniae*
18	*Enterococcus saccharolyticus*	21	*Enterococcus saccharolyticus*
12	*Clostridium septicum*	17	*Klebsiella oxytoca*

11	*Corynebacterium simulans*	15	*Corynebacterium striatum*
10	*Shigella sonnei*	13	*Staphylococcus caprae*
10	*Eikenella corrodens*	13	*Ochrobactrum anthropi*
9	*Staphylococcus sciuri*	13	*Eikenella corrodens*
9	*Bacillus amyloliquefaciens*	12	*Staphylococcus sciuri*
8	*Ochrobactrum anthropi*	12	*Clostridium septicum*
7	*Proteus mirabilis*	10	*[Clostridium] sordellii*
7	*Aggregatibacter aphrophilus*	9	*Aggregatibacter aphrophilus*
6	*Staphylococcus epidermidis*	8	*Enterococcus faecium*
6	*Staphylococcus aureus*	7	*Proteus mirabilis*
6	*Oligella ureolytica*	7	*Oligella ureolytica*
6	*Neisseria gonorrhoeae*	7	*Escherichia coli*
6	*Enterococcus faecium*	7	*Bacteroides ovatus*
6	*Enterococcus casseliflavus*	6	*Staphylococcus aureus*
5	*Stenotrophomonas maltophilia*	6	*Proteus vulgaris*
5	*Pseudomonas aeruginosa*	6	*Neisseria gonorrhoeae*
5	*Proteus vulgaris*	6	*Haemophilus influenzae*
4	*Streptococcus thermophilus*	6	*Enterobacteriaceae bacterium*
3	*Staphylococcus saprophyticus*	5	*Streptococcus salivarius*
3	*Staphylococcus caprae*	5	*Stenotrophomonas maltophilia*
3	*Propionibacterium acnes*	5	*Pseudomonas aeruginosa*
3	*Haemophilus influenzae*	5	*Paenibacillus polymyxa*
3	*Enterococcus camelliae*	5	*Enterococcus casseliflavus*
3	*[Clostridium] sordellii*	4	*Spirosoma rigui*
3	*Bacteroides ovatus*	4	*Spirosoma linguale*
2	*Haemophilus aegyptius*	3	*Staphylococcus saprophyticus*
2	*Clostridium perfringens*	3	*Propionibacterium acnes*
1	*Streptococcus dysgalactiae*	3	*Lactococcus lactis*
1	*Staphylococcus saccharolyticus*	2	*Xanthomonadales bacterium*
1	*Staphylococcus hominis*	2	*Enterococcus gallinarum*
1	*Shigella flexneri*	2	*Enterococcus faecalis*
1	*Shigella dysenteriae*	2	*Enterobacter cloacae*
1	*Shigella boydii*	2	*Clostridium perfringens*
1	*Salmonella subterranea*	1	*Staphylococcus saccharolyticus*
1	*Paenibacillus jamilae*	1	*Staphylococcus hominis*
1	*Micrococcus luteus*	1	*Shigella sonnei*
1	*Lactococcus lactis*	1	*Shigella boydii*
1	*Kocuria kristinae*	1	*Serratia grimesii*
1	*Escherichia fergusonii*	1	*Pontibacter indicus*
1	*Enterococcus faecalis*	1	*Micrococcus luteus*
1	*Enterococcus durans*	1	*Kocuria rosea*
1	*Bacillus subtilis*	1	*Fusobacterium periodonticum*
1	*Atopobium parvulum*	1	*Fluviicola taffensis*
45108	Total Reads	1	*Enterococcus hirae*
		1	*Enterobacter hormaechei*
		1	*Empedobacter falsenii*
		1	*Corynebacterium renale*

		1	*Citrobacter freundii*
		1	*Atopobium parvulum*
		1	*Anaerococcus obesiensis*
		49217	Total Reads
Organism sequenced: *Enterococcus saccharolyticus* ATCC 43076			
blastn number of reads matched	blastn organism matched	SequenceMatch number of reads matched	SequenceMatch organism matched
136113	*Enterococcus saccharolyticus*	158573	*Enterococcus saccharolyticus*
1079	*Bacillus vietnamensis*	63	*Listeria monocytogenes*
258	*Gracilibacillus dipsosauri*	36	*Klebsiella oxytoca*
45	*Bacillus megaterium*	19	*Neisseria gonorrhoeae*
36	*Klebsiella oxytoca*	18	*Paenibacillus polymyxa*
32	*Listeria monocytogenes*	17	*Streptococcus pneumoniae*
15	*Neisseria gonorrhoeae*	15	*Ochrobactrum anthropi*
14	*Streptococcus pneumoniae*	14	*Staphylococcus sciuri*
12	*Shigella sonnei*	14	*[Clostridium] sordellii*
11	*Enterococcus camelliae*	11	*Enterococcus faecium*
11	*Aggregatibacter aphrophilus*	11	*Bacteroides ovatus*
10	*Ochrobactrum anthropi*	11	*Aggregatibacter aphrophilus*
10	*Enterococcus faecium*	10	*Pseudomonas aeruginosa*
10	*Enterococcus casseliflavus*	9	*Streptococcus pyogenes*
9	*Staphylococcus epidermidis*	9	*Stenotrophomonas maltophilia*
9	*Paenibacillus polymyxa*	9	*Staphylococcus epidermidis*
8	*Streptococcus pyogenes*	9	*Staphylococcus aureus*
8	*Staphylococcus sciuri*	9	*Eikenella corrodens*
8	*Pseudomonas aeruginosa*	8	*Shigella sonnei*
8	*Clostridium septicum*	8	*Proteus vulgaris*
7	*Streptococcus thermophilus*	8	*Proteus mirabilis*
7	*Stenotrophomonas maltophilia*	8	*Escherichia coli*
6	*Staphylococcus aureus*	8	*Clostridium septicum*
6	*Proteus vulgaris*	7	*Enterococcus casseliflavus*
6	*Proteus mirabilis*	7	*Elizabethkingia meningoseptica*
6	*Elizabethkingia meningoseptica*	7	*Corynebacterium striatum*
6	*Eikenella corrodens*	6	*Streptococcus salivarius*
6	*Corynebacterium simulans*	6	*Haemophilus influenzae*
6	*[Clostridium] sordellii*	6	*Enterococcus gallinarum*
6	*Clostridium perfringens*	6	*Enterococcus faecalis*
5	*Oligella ureolytica*	5	*Streptococcus equi*
5	*Bacteroides ovatus*	5	*Staphylococcus saprophyticus*
4	*Streptococcus equi*	5	*Oligella ureolytica*
4	*Staphylococcus saprophyticus*	5	*Kocuria rosea*
4	*Shigella flexneri*	5	*Enterobacteriaceae bacterium*
4	*Paenibacillus jamilae*	5	*Clostridium perfringens*
3	*Shigella boydii*	3	*Serratia proteamaculans*
3	*Serratia grimesii*	3	*Enterobacter hormaechei*

3	*Haemophilus influenzae*	2	*Shigella flexneri*
3	*Enterococcus faecalis*	2	*Ochrobactrum cytisi*
2	*Shigella dysenteriae*	1	*Vibrio fluvialis*
2	*Pseudomonas entomophila*	1	*Shigella boydii*
2	*Kocuria kristinae*	1	*Pseudomonas tolaasii*
2	*Bacillus subtilis*	1	*Pseudomonas putida*
2	*Bacillus pseudalcaliphilus*	1	*Pseudomonas mevalonii*
1	*Streptococcus mitis*	1	*Paenibacillus jamilae*
1	*Serratia proteamaculans*	1	*Geobacillus toebii*
1	*Pseudomonas tolaasii*	1	*Clostridiales bacterium*
1	*Proteus hauseri*	1	*Citrobacter freundii*
1	*Geobacillus galactosidasius*	1	*Aeromonas punctata*
1	*Citrobacter koseri*	1	*Aeromonas caviae*
1	*Bacillus fastidiosus*	158993	Total Reads
1	*Aeromonas hydrophila*		
137824	Total Reads		

Organism sequenced: *Enterococcus casseliflavus* ATCC 700327			
blastn number of reads matched	blastn organism matched	SequenceMatch number of reads matched	SequenceMatch organism matched
51029	*Enterococcus casseliflavus*	36220	*Enterococcus casseliflavus*
151	*Bacillus vietnamensis*	16329	*Enterococcus gallinarum*
102	*Bacillus subtilis*	5604	*Enterococcus faecalis*
64	*Enterococcus gallinarum*	674	*Vibrio fluvialis*
20	*Listeria monocytogenes*	28	*Listeria monocytogenes*
13	*Klebsiella oxytoca*	15	*Klebsiella oxytoca*
11	*Enterococcus saccharolyticus*	13	*Enterococcus saccharolyticus*
9	*Staphylococcus sciuri*	11	*[Clostridium] sordellii*
9	*Shigella sonnei*	10	*Proteus mirabilis*
9	*Proteus mirabilis*	10	*Neisseria gonorrhoeae*
8	*Streptococcus pneumoniae*	9	*Staphylococcus sciuri*
8	*Neisseria gonorrhoeae*	9	*Enterobacteriaceae bacterium*
7	*[Clostridium] sordellii*	8	*Streptococcus pneumoniae*
6	*Gracilibacillus dipsosauri*	7	*Ochrobactrum anthropi*
5	*Ochrobactrum anthropi*	7	*Corynebacterium striatum*
5	*Eikenella corrodens*	5	*Pseudomonas aeruginosa*
5	*Corynebacterium simulans*	5	*Eikenella corrodens*
5	*Aggregatibacter aphrophilus*	5	*Aggregatibacter aphrophilus*
4	*Pseudomonas aeruginosa*	4	*Streptococcus pyogenes*
4	*Oligella ureolytica*	4	*Paenibacillus polymyxa*
4	*Enterococcus faecalis*	4	*Oligella ureolytica*
4	*Enterococcus camelliae*	4	*Enterococcus faecium*
4	*Clostridium septicum*	4	*Clostridium septicum*
4	*Bacillus megaterium*	3	*Stenotrophomonas maltophilia*
3	*Streptococcus pyogenes*	3	*Staphylococcus aureus*
3	*Staphylococcus aureus*	3	*Kocuria rosea*

3	*Kocuria kristinae*	3	*Haemophilus influenzae*
3	*Bacillus pseudalcaliphilus*	3	*Escherichia coli*
2	*Streptococcus thermophilus*	3	*Clostridium perfringens*
2	*Stenotrophomonas maltophilia*	2	*Streptococcus salivarius*
2	*Proteus vulgaris*	2	*Streptococcus equi*
2	*Paenibacillus jamilae*	2	*Shigella boydii*
2	*Haemophilus aegyptius*	2	*Proteus vulgaris*
2	*Enterococcus hirae*	2	*Elizabethkingia meningoseptica*
2	*Enterococcus faecium*	1	*Staphylococcus epidermidis*
2	*Bacillus tequilensis*	1	*Shigella flexneri*
1	*Streptococcus equi*	1	*Enterococcus hirae*
1	*Staphylococcus epidermidis*	1	*Enterococcus durans*
1	*Shigella boydii*	1	*Citrobacter freundii*
1	*Proteus hauseri*	1	*Bacteroides ovatus*
1	*Escherichia fergusonii*	59023	Total Reads
1	*Enterococcus thailandicus*		
1	*Enterococcus durans*		
1	*Elizabethkingia meningoseptica*		
1	*Clostridium perfringens*		
1	*Bacteroides ovatus*		
51528	Total Reads		

Organism sequenced: *Staphylococcus saprophyticus* BAA 750			
blastn number of reads matched	blastn organism matched	SequenceMatch number of reads matched	SequenceMatch organism matched
23359	*Staphylococcus saprophyticus*	23402	*Staphylococcus saprophyticus*
17	*Listeria monocytogenes*	198	*Staphylococcus xylosus*
12	*Klebsiella oxytoca*	28	*Listeria monocytogenes*
11	*Streptococcus pneumoniae*	14	*Klebsiella oxytoca*
10	*[Clostridium] sordellii*	13	*Streptococcus pneumoniae*
9	*Neisseria gonorrhoeae*	13	*[Clostridium] sordellii*
9	*Clostridium septicum*	13	*Bacteroides ovatus*
8	*Staphylococcus aureus*	11	*Staphylococcus aureus*
7	*Staphylococcus sciuri*	10	*Staphylococcus sciuri*
7	*Pseudomonas aeruginosa*	10	*Paenibacillus polymyxa*
7	*Enterococcus faecalis*	10	*Enterococcus saccharolyticus*
7	*Bacteroides ovatus*	10	*Clostridium septicum*
6	*Shigella sonnei*	9	*Staphylococcus cohnii*
6	*Enterococcus saccharolyticus*	9	*Neisseria gonorrhoeae*
5	*Paenibacillus polymyxa*	8	*Pseudomonas aeruginosa*
5	*Enterococcus casseliflavus*	7	*Enterococcus faecalis*
4	*Streptococcus pyogenes*	6	*Proteus vulgaris*
4	*Staphylococcus epidermidis*	5	*Staphylococcus epidermidis*
4	*Proteus vulgaris*	5	*Escherichia coli*
4	*Proteus mirabilis*	4	*Streptococcus pyogenes*
3	*Paenibacillus jamilae*	4	*Shigella sonnei*

3	*Ochrobactrum anthropi*	4	*Proteus mirabilis*
3	*Enterococcus faecium*	4	*Enterococcus faecium*
3	*Enterococcus camelliae*	4	*Enterococcus casseliflavus*
3	*Aggregatibacter aphrophilus*	3	*Streptococcus equi*
2	*Streptococcus equi*	3	*Ochrobactrum anthropi*
2	*Stenotrophomonas maltophilia*	3	*Haemophilus influenzae*
2	*Shigella flexneri*	3	*Enterobacteriaceae bacterium*
2	*Shigella dysenteriae*	3	*Aggregatibacter aphrophilus*
2	*Oligella ureolytica*	2	*Stenotrophomonas maltophilia*
2	*Haemophilus aegyptius*	2	*Oligella ureolytica*
2	*Escherichia fergusonii*	2	*Enterococcus gallinarum*
1	*Proteus hauseri*	2	*Enterobacter hormaechei*
1	*Kocuria kristinae*	2	*Corynebacterium striatum*
1	*Haemophilus influenzae*	1	*Staphylococcus warneri*
1	*Enterococcus sulfureus*	1	*Staphylococcus devriesei*
1	*Clostridium perfringens*	1	*Proteus hauseri*
1	*Citrobacter koseri*	1	*Ochrobactrum cytisi*
1	*Bacillus siralis*	1	*Kocuria rosea*
23537	Total Reads	1	*Clostridium perfringens*
		1	*Aeromonas veronii*
		23833	Total Reads

Organism sequenced: *Listeria monocytogenes* BAA 751			
blastn number of reads matched	blastn organism matched	SequenceMatch number of reads matched	SequenceMatch organism matched
33321	*Listeria monocytogenes*	57676	*Listeria monocytogenes*
5040	*Enterococcus camelliae*	40	*Klebsiella oxytoca*
179	*Enterococcus sulfureus*	38	*Enterococcus saccharolyticus*
71	*Listeria innocua*	24	*Paenibacillus polymyxa*
37	*Klebsiella oxytoca*	23	*Streptococcus pneumoniae*
31	*Enterococcus saccharolyticus*	18	*[Clostridium] sordellii*
21	*Shigella sonnei*	16	*Streptococcus pyogenes*
16	*Streptococcus pneumoniae*	16	*Proteus mirabilis*
13	*Neisseria gonorrhoeae*	15	*Staphylococcus sciuri*
13	*Aggregatibacter aphrophilus*	15	*Neisseria gonorrhoeae*
12	*Staphylococcus epidermidis*	14	*Staphylococcus aureus*
12	*Proteus mirabilis*	14	*Bacteroides ovatus*
11	*Streptococcus pyogenes*	14	*Aggregatibacter aphrophilus*
11	*Staphylococcus sciuri*	13	*Shigella sonnei*
11	*Clostridium septicum*	13	*Ochrobactrum anthropi*
10	*Staphylococcus aureus*	13	*Eikenella corrodens*
10	*Proteus vulgaris*	13	*Clostridium septicum*
10	*[Clostridium] sordellii*	12	*Proteus vulgaris*
9	*Paenibacillus polymyxa*	12	*Corynebacterium striatum*
9	*Ochrobactrum anthropi*	11	*Staphylococcus epidermidis*

9	*Enterococcus casseliflavus*	11	*Enterobacteriaceae bacterium*
9	*Eikenella corrodens*	9	*Clostridium perfringens*
8	*Bacteroides ovatus*	8	*Enterococcus faecium*
7	*Streptococcus equi*	8	*Enterococcus faecalis*
7	*Stenotrophomonas maltophilia*	7	*Streptococcus equi*
7	*Corynebacterium simulans*	7	*Stenotrophomonas maltophilia*
7	*Clostridium perfringens*	7	*Escherichia coli*
6	*Enterococcus faecium*	7	*Enterococcus casseliflavus*
5	*Streptococcus thermophilus*	6	*Haemophilus influenzae*
5	*Oligella ureolytica*	5	*Streptococcus salivarius*
5	*Enterococcus faecalis*	5	*Oligella ureolytica*
4	*Shigella flexneri*	4	*Enterobacter hormaechei*
4	*Paenibacillus jamilae*	3	*Staphylococcus saprophyticus*
3	*Staphylococcus saprophyticus*	3	*Shigella flexneri*
3	*Shigella boydii*	3	*Citrobacter freundii*
3	*Citrobacter koseri*	2	*Pseudomonas aeruginosa*
2	*Pseudomonas aeruginosa*	2	*Listeria welshimeri*
2	*Haemophilus influenzae*	2	*Listeria innocua*
1	*Shigella dysenteriae*	2	*Kocuria rosea*
1	*Proteus hauseri*	2	*Enterococcus gallinarum*
1	*Haemophilus aegyptius*	2	*Elizabethkingia meningoseptica*
1	*Gracilibacillus dipsosauri*	1	*Shigella boydii*
1	*Escherichia fergusonii*	1	*Enterobacter ludwigii*
1	*Elizabethkingia meningoseptica*	58117	Total Reads
1	*Clostridium hydrogeniformans*		
1	*Bacillus vietnamensis*		
1	*Bacillus fordii*		
38952	Total Reads		

Organism sequenced: *Streptococcus pneumoniae* ATCC 49619			
blastn number of reads matched	blastn organism matched	SequenceMatch number of reads matched	SequenceMatch organism matched
41080	*Streptococcus pneumoniae*	46839	*Streptococcus pneumoniae*
330	*Streptococcus mitis*	40	*Streptococcus mitis*
27	*Streptococcus pseudopneumoniae*	16	*Listeria monocytogenes*
11	*Listeria monocytogenes*	11	*Enterococcus saccharolyticus*
10	*Enterococcus saccharolyticus*	9	*Streptococcus equi*
8	*Streptococcus equi*	8	*Streptococcus pyogenes*
7	*Klebsiella oxytoca*	7	*Proteus vulgaris*
6	*Streptococcus pyogenes*	7	*Klebsiella oxytoca*
6	*Proteus vulgaris*	5	*Pseudomonas aeruginosa*
5	*Pseudomonas aeruginosa*	5	*Proteus mirabilis*
5	*Proteus mirabilis*	5	*Ochrobactrum anthropi*
4	*Shigella sonnei*	5	*Bacteroides ovatus*
4	*Clostridium perfringens*	4	*Clostridium perfringens*

3	*Ochrobactrum anthropi*	3	*Shigella sonnei*
3	*Neisseria gonorrhoeae*	3	*Neisseria gonorrhoeae*
3	*Enterococcus casseliflavus*	3	*Eikenella corrodens*
3	*Enterococcus camelliae*	2	*Streptococcus salivarius*
3	*Eikenella corrodens*	2	*Staphylococcus aureus*
3	*Bacteroides ovatus*	2	*Shigella flexneri*
2	*Streptococcus thermophilus*	2	*Paenibacillus jamilae*
2	*Oligella ureolytica*	2	*Oligella ureolytica*
2	*Enterococcus faecium*	2	*Enterococcus gallinarum*
2	*Citrobacter koseri*	2	*Enterococcus faecium*
2	*Aggregatibacter aphrophilus*	2	*Enterococcus casseliflavus*
1	*Streptococcus oralis*	2	*Corynebacterium striatum*
1	*Staphylococcus sciuri*	2	*[Clostridium] sordellii*
1	*Staphylococcus epidermidis*	2	*Aggregatibacter aphrophilus*
1	*Staphylococcus aureus*	1	*Streptococcus pseudopneumoniae*
1	*Shigella boydii*	1	*Stenotrophomonas maltophilia*
1	*Paenibacillus polymyxa*	1	*Staphylococcus sciuri*
1	*Paenibacillus jamilae*	1	*Staphylococcus epidermidis*
1	*Enterococcus faecalis*	1	*Shigella boydii*
1	*Corynebacterium simulans*	1	*Kocuria rosea*
1	*[Clostridium] sordellii*	1	*Haemophilus influenzae*
1	*Clostridium septicum*	1	*Escherichia coli*
41542	Total Reads	1	*Enterococcus faecalis*
		1	*Enterobacteriaceae bacterium*
		1	*Clostridium septicum*
		47003	Total Reads

Organism sequenced: *Staphylococcus sciuri* ATCC 29061			
blastn number of reads matched	blastn organism matched	SequenceMatch number of reads matched	SequenceMatch organism matched
58120	*Staphylococcus sciuri*	67803	*Staphylococcus sciuri*
23	*Listeria monocytogenes*	41	*Listeria monocytogenes*
22	*Klebsiella oxytoca*	26	*Klebsiella oxytoca*
14	*Bacteroides ovatus*	18	*Bacteroides ovatus*
11	*Neisseria gonorrhoeae*	13	*Staphylococcus aureus*
10	*Streptococcus pneumoniae*	13	*Enterococcus saccharolyticus*
9	*Shigella sonnei*	12	*Neisseria gonorrhoeae*
8	*Pseudomonas aeruginosa*	12	*[Clostridium] sordellii*
7	*Stenotrophomonas maltophilia*	11	*Streptococcus pneumoniae*
7	*Enterococcus saccharolyticus*	9	*Shigella sonnei*
6	*Streptococcus thermophilus*	9	*Pseudomonas aeruginosa*
6	*Streptococcus pyogenes*	9	*Paenibacillus polymyxa*
6	*Staphylococcus aureus*	7	*Streptococcus pyogenes*
6	*Paenibacillus polymyxa*	7	*Stenotrophomonas maltophilia*
6	*[Clostridium] sordellii*	6	*Streptococcus salivarius*
5	*Enterococcus camelliae*	6	*Enterobacteriaceae bacterium*

5	*Eikenella corrodens*	6	*Eikenella corrodens*
4	*Proteus vulgaris*	6	*Clostridium septicum*
4	*Clostridium septicum*	5	*Staphylococcus stepanovicii*
3	*Staphylococcus saprophyticus*	5	*Proteus vulgaris*
3	*Enterococcus faecium*	5	*Proteus mirabilis*
3	*Citrobacter koseri*	4	*Ochrobactrum anthropi*
3	*Aggregatibacter aphrophilus*	4	*Enterococcus faecium*
2	*Staphylococcus epidermidis*	3	*Staphylococcus saprophyticus*
2	*Proteus mirabilis*	3	*Haemophilus influenzae*
2	*Proteus hauseri*	3	*Corynebacterium striatum*
2	*Ochrobactrum anthropi*	3	*Aggregatibacter aphrophilus*
2	*Elizabethkingia meningoseptica*	2	*Staphylococcus vitulinus*
2	*Corynebacterium simulans*	2	*Staphylococcus epidermidis*
1	*Shigella flexneri*	2	*Serratia proteamaculans*
1	*Shigella boydii*	2	*Escherichia coli*
1	*Serratia proteamaculans*	2	*Elizabethkingia meningoseptica*
1	*Serratia grimesii*	1	*Staphylococcus lentus*
1	*Paenibacillus jamilae*	1	*Shigella flexneri*
1	*Oligella ureolytica*	1	*Shigella boydii*
1	*Lysinibacillus xylanilyticus*	1	*Oligella ureolytica*
1	*Listeria innocua*	1	*Listeria welshimeri*
1	*Haemophilus influenzae*	1	*Kocuria rosea*
1	*Haemophilus aegyptius*	1	*Enterococcus faecalis*
1	*Escherichia fergusonii*	1	*Enterococcus casseliflavus*
1	*Enterococcus faecalis*	1	*Enterobacter hormaechei*
1	*Enterococcus casseliflavus*	1	*Clostridium perfringens*
1	*Clostridium perfringens*	1	*Citrobacter freundii*
58317	Total Reads	68070	Total Reads

Organism sequenced: *Klebsiella oxytoca* ATCC 700324

blastn number of reads matched	blastn organism matched	SequenceMatch number of reads matched	SequenceMatch organism matched
72674	*Klebsiella oxytoca*	77462	*Klebsiella oxytoca*
39	*Citrobacter freundii*	3462	*Citrobacter freundii*
29	*Shigella sonnei*	21	*Listeria monocytogenes*
17	*Escherichia coli*	16	*Shigella sonnei*
15	*Listeria monocytogenes*	12	*Enterobacteriaceae bacterium*
10	*Neisseria gonorrhoeae*	11	*Neisseria gonorrhoeae*
7	*Enterococcus saccharolyticus*	11	*Haemophilus influenzae*
6	*Stenotrophomonas maltophilia*	10	*Escherichia coli*
6	*Proteus vulgaris*	10	*Bacteroides ovatus*
5	*Streptococcus pyogenes*	8	*Stenotrophomonas maltophilia*
5	*Shigella flexneri*	8	*Proteus vulgaris*
5	*Oligella ureolytica*	8	*Ochrobactrum anthropi*
4	*Streptococcus thermophilus*	7	*Streptococcus pyogenes*

4	*Streptococcus pneumoniae*	7	*Proteus mirabilis*
4	*Pseudomonas aeruginosa*	7	*Enterococcus saccharolyticus*
4	*Proteus mirabilis*	7	*Aggregatibacter aphrophilus*
4	*Ochrobactrum anthropi*	6	*Oligella ureolytica*
4	*Haemophilus aegyptius*	6	*Grimontella senegalensis*
4	*Aggregatibacter aphrophilus*	5	*Streptococcus pneumoniae*
3	*Haemophilus influenzae*	5	*Pseudomonas aeruginosa*
3	*Eikenella corrodens*	5	*Enterococcus faecalis*
3	*Bacteroides ovatus*	5	*[Clostridium] sordellii*
2	*Streptococcus mitis*	5	*Citrobacter braakii*
2	*Staphylococcus sciuri*	4	*Streptococcus salivarius*
2	*Staphylococcus aureus*	4	*Paenibacillus polymyxa*
2	*Shigella boydii*	4	*Eikenella corrodens*
2	*Enterococcus faecalis*	3	*Enterobacter hormaechei*
2	*[Clostridium] sordellii*	3	*Clostridium septicum*
2	*Clostridium septicum*	2	*Staphylococcus sciuri*
1	*Streptococcus equi*	2	*Staphylococcus aureus*
1	*Staphylococcus epidermidis*	2	*Shigella flexneri*
1	*Shigella dysenteriae*	2	*Enterococcus faecium*
1	*Paenibacillus polymyxa*	2	*Enterococcus casseliflavus*
1	*Listeria innocua*	2	*Corynebacterium striatum*
1	*Escherichia fergusonii*	2	*Citrobacter werkmanii*
1	*Erwinia amylovora*	1	*Streptococcus equi*
1	*Enterococcus hirae*	1	*Staphylococcus epidermidis*
1	*Enterococcus faecium*	1	*Leclercia adecarboxylata*
1	*Enterococcus casseliflavus*	1	*Escherichia fergusonii*
1	*Enterococcus camelliae*	1	*Enterococcus gallinarum*
1	*Enterobacter mori*	1	*Elizabethkingia meningoseptica*
1	*Corynebacterium simulans*	1	*Clostridium perfringens*
1	*Clostridium perfringens*	81143	Total Reads
1	*Citrobacter koseri*		
72884	Total Reads		

Organism sequenced: *Stenotrophomonas maltophilia* ATCC 17666

blastn number of reads matched	blastn organism matched	SequenceMatch number of reads matched	SequenceMatch organism matched
42111	*Stenotrophomonas maltophilia*	49087	*Stenotrophomonas maltophilia*
28	*Klebsiella oxytoca*	29	*Klebsiella oxytoca*
13	*Shigella sonnei*	19	*Bacteroides ovatus*
12	*Oligella ureolytica*	14	*Neisseria gonorrhoeae*
12	*Bacteroides ovatus*	13	*Listeria monocytogenes*
11	*Neisseria gonorrhoeae*	12	*Oligella ureolytica*
8	*Aggregatibacter aphrophilus*	11	*Haemophilus influenzae*
7	*Listeria monocytogenes*	10	*Aggregatibacter aphrophilus*
6	*Streptococcus pyogenes*	9	*Streptococcus pyogenes*

6	*Staphylococcus sciuri*	9	*Eikenella corrodens*
6	*Pseudomonas aeruginosa*	8	*Pseudomonas aeruginosa*
6	*Proteus mirabilis*	7	*Shigella sonnei*
6	*Enterococcus saccharolyticus*	7	*Proteus mirabilis*
5	*Shigella flexneri*	7	*Escherichia coli*
5	*Eikenella corrodens*	7	*Enterococcus saccharolyticus*
4	*Streptococcus thermophilus*	6	*Staphylococcus sciuri*
4	*Haemophilus aegyptius*	5	*Paenibacillus polymyxa*
3	*Stenotrophomonas chelatiphaga*	4	*Streptococcus salivarius*
3	*Shigella dysenteriae*	4	*Streptococcus pneumoniae*
3	*Shigella boydii*	4	*Enterobacteriaceae bacterium*
3	*Enterococcus camelliae*	4	*Enterobacter hormaechei*
2	*Streptococcus equi*	4	*Elizabethkingia meningoseptica*
2	*Staphylococcus aureus*	4	*[Clostridium] sordellii*
2	*Paenibacillus polymyxa*	3	*Streptococcus equi*
2	*Haemophilus influenzae*	3	*Shigella flexneri*
2	*Escherichia fergusonii*	3	*Enterococcus faecalis*
2	*Elizabethkingia meningoseptica*	3	*Clostridium septicum*
2	*Corynebacterium simulans*	3	*Citrobacter freundii*
2	*Clostridium septicum*	2	*Staphylococcus aureus*
2	*Clostridium perfringens*	2	*Shigella boydii*
1	*Streptococcus pneumoniae*	2	*Proteus vulgaris*
1	*Proteus vulgaris*	2	*Ochrobactrum anthropi*
1	*Paenibacillus jamilae*	2	*Enterococcus casseliflavus*
1	*Enterococcus faecalis*	2	*Corynebacterium striatum*
1	*Enterococcus casseliflavus*	2	*Clostridium perfringens*
1	*[Clostridium] sordellii*	1	*Proteus hauseri*
1	*Clostridium hydrogeniformans*	1	*Enterobacter ludwigii*
42287	Total Reads	49315	Total Reads

Organism sequenced: *Elizabethkingia meningoseptica* ATCC 13253			
blastn number of reads matched	blastn organism matched	SequenceMatch number of reads matched	SequenceMatch organism matched
19378	*Elizabethkingia meningoseptica*	25931	*Elizabethkingia meningoseptica*
13	*Klebsiella oxytoca*	14	*Klebsiella oxytoca*
5	*Proteus mirabilis*	6	*Bacteroides ovatus*
4	*Shigella sonnei*	5	*Proteus mirabilis*
4	*Pseudomonas aeruginosa*	4	*Pseudomonas aeruginosa*
4	*Enterococcus saccharolyticus*	4	*Listeria monocytogenes*
3	*Shigella dysenteriae*	4	*Escherichia coli*
3	*Listeria monocytogenes*	4	*Enterococcus saccharolyticus*
3	*[Clostridium] sordellii*	3	*Streptococcus pyogenes*
3	*Clostridium septicum*	3	*Proteus vulgaris*
3	*Bacteroides ovatus*	3	*Enterobacteriaceae bacterium*
2	*Streptococcus thermophilus*	3	*[Clostridium] sordellii*

2	*Staphylococcus saprophyticus*	3	*Clostridium septicum*
2	*Proteus vulgaris*	2	*Streptococcus salivarius*
2	*Enterococcus faecalis*	2	*Staphylococcus saprophyticus*
2	*Enterococcus casseliflavus*	2	*Shigella sonnei*
1	*Streptococcus pyogenes*	2	*Enterococcus faecalis*
1	*Streptococcus pneumoniae*	2	*Citrobacter freundii*
1	*Stenotrophomonas maltophilia*	1	*Vibrio fluvialis*
1	*Staphylococcus sciuri*	1	*Streptococcus pneumoniae*
1	*Shigella boydii*	1	*Stenotrophomonas maltophilia*
1	*Paenibacillus jamilae*	1	*Staphylococcus sciuri*
1	*Oligella ureolytica*	1	*Staphylococcus aureus*
1	*Ochrobactrum anthropi*	1	*Shigella boydii*
1	*Neisseria gonorrhoeae*	1	*Paenibacillus polymyxa*
1	*Eikenella corrodens*	1	*Oligella ureolytica*
1	*Corynebacterium simulans*	1	*Ochrobactrum cytisi*
1	*Clostridium perfringens*	1	*Neisseria gonorrhoeae*
1	*Bacillus halodurans*	1	*Enterococcus casseliflavus*
19446	Total Reads	1	*Enterobacter ludwigii*
		1	*Eikenella corrodens*
		1	*Corynebacterium striatum*
		1	*Clostridium perfringens*
		26012	Total Reads

Organism sequenced: *Corynebacterium striatum* BAA 1293

blastn number of reads matched	blastn organism matched	SequenceMatch number of reads matched	SequenceMatch organism matched
23515	*Corynebacterium simulans*	34843	*Corynebacterium striatum*
79	*Pseudomonas cedrina*	41	*Pseudomonas veronii*
23	*Klebsiella oxytoca*	27	*Pseudomonas fluorescens*
10	*Listeria monocytogenes*	22	*Klebsiella oxytoca*
10	*Enterococcus casseliflavus*	16	*Listeria monocytogenes*
8	*Streptococcus pneumoniae*	11	*Enterococcus saccharolyticus*
8	*Staphylococcus sciuri*	10	*Pseudomonas synxantha*
8	*Enterococcus saccharolyticus*	9	*Streptococcus pneumoniae*
7	*Oligella ureolytica*	9	*Staphylococcus sciuri*
7	*Neisseria gonorrhoeae*	9	*Escherichia coli*
6	*Pantoea vagans*	9	*Bacteroides ovatus*
6	*Bacteroides ovatus*	8	*Oligella ureolytica*
5	*Enterococcus faecium*	8	*Neisseria gonorrhoeae*
5	*Eikenella corrodens*	7	*Corynebacterium xerosis*
4	*Shigella sonnei*	6	*Ochrobactrum anthropi*
4	*Shigella dysenteriae*	6	*Enterococcus casseliflavus*
4	*Clostridium perfringens*	6	*Enterobacteriaceae bacterium*
4	*Aeromonas hydrophila*	5	*Haemophilus influenzae*
3	*Streptococcus pyogenes*	5	*Enterococcus faecium*

3	*Proteus mirabilis*	5	*Enterococcus faecalis*
3	*Ochrobactrum anthropi*	5	*Eikenella corrodens*
3	*Haemophilus aegyptius*	4	*Streptococcus pyogenes*
3	*Aggregatibacter aphrophilus*	4	*Sphingomonas dokdonensis*
2	*Streptococcus thermophilus*	4	*Pseudomonas trivialis*
2	*Staphylococcus saprophyticus*	4	*Proteus mirabilis*
2	*Staphylococcus epidermidis*	4	*Clostridium perfringens*
2	*Staphylococcus aureus*	4	*Aeromonas punctata*
2	*Pantoea allii*	3	*Staphylococcus aureus*
2	*Enterococcus faecalis*	3	*Pantoea agglomerans*
2	*Clostridium septicum*	3	*Paenibacillus polymyxa*
2	*Acidovorax ebreus*	3	*Moraxella osloensis*
1	*Streptococcus equi*	3	*Flavobacteriaceae bacterium*
1	*Stenotrophomonas maltophilia*	3	*Aggregatibacter aphrophilus*
1	*Pseudomonas tolaasii*	2	*Xanthomonadales bacterium*
1	*Pseudomonas palleroniana*	2	*Streptococcus salivarius*
1	*Pseudomonas jessenii*	2	*Staphylococcus saprophyticus*
1	*Pseudomonas grimontii*	2	*Staphylococcus epidermidis*
1	*Pseudomonas aeruginosa*	2	*Sphingomonas yunnanensis*
1	*Proteus vulgaris*	2	*Shigella boydii*
1	*Proteus hauseri*	2	*Pseudomonas chlororaphis*
1	*Paenibacillus polymyxa*	2	*Clostridium septicum*
1	*Moraxella osloensis*	2	*Candidatus Roseomonas*
1	*Haemophilus influenzae*	2	*Acidovorax ebreus*
1	*Enterococcus camelliae*	1	*Yersinia mollaretii*
1	*Elizabethkingia meningoseptica*	1	*Streptococcus equi*
1	*Chryseobacterium gleum*	1	*Stenotrophomonas maltophilia*
1	*Aeromonas media*	1	*Sphingomonas azotifigens*
23760	Total Reads	1	*Shigella sonnei*
		1	*Rahnella aquatilis*
		1	*Pseudomonas palleroniana*
		1	*Pseudomonas aeruginosa*
		1	*Proteus vulgaris*
		1	*Naumannella halotolerans*
		1	*Kocuria rhizophila*
		1	*Enterococcus gallinarum*
		1	*Enterobacter ludwigii*
		1	*Enterobacter hormaechei*
		1	*Enterobacter cloacae*
		1	*Empedobacter falsenii*
		1	*Elizabethkingia meningoseptica*
		1	*[Clostridium] sordellii*
		1	*Citrobacter freundii*
		1	*Chryseobacterium gleum*
		1	*Bdellovibrio bacteriovorus*
		1	*Aeromonas media*
		35151	Total Reads

Organism sequenced: *Paenibacillus polymyxa* ATCC 7070			
blastn number of reads matched	blastn organism matched	SequenceMatch number of reads matched	SequenceMatch organism matched
11705	*Paenibacillus polymyxa*	23248	*Paenibacillus polymyxa*
3937	*Paenibacillus jamilae*	819	*Paenibacillus jamilae*
232	*Clostridium hydrogeniformans*	40	*Listeria monocytogenes*
45	*Pullulanibacillus naganoensis*	22	*Klebsiella oxytoca*
25	*Listeria monocytogenes*	20	*Enterococcus saccharolyticus*
20	*Klebsiella oxytoca*	16	*Streptococcus pneumoniae*
17	*Enterococcus saccharolyticus*	16	*Staphylococcus sciuri*
15	*Shigella sonnei*	11	*[Clostridium] sordellii*
14	*Staphylococcus sciuri*	10	*Shigella sonnei*
12	*Streptococcus pneumoniae*	10	*Proteus mirabilis*
10	*Neisseria gonorrhoeae*	10	*Neisseria gonorrhoeae*
8	*Proteus mirabilis*	8	*Proteus vulgaris*
7	*Paenibacillus peoriae*	8	*Haemophilus influenzae*
7	*Enterococcus casseliflavus*	8	*Corynebacterium striatum*
6	*Proteus vulgaris*	8	*Clostridium septicum*
6	*Oligella ureolytica*	7	*Streptococcus pyogenes*
6	*[Clostridium] sordellii*	7	*Bacteroides ovatus*
6	*Clostridium septicum*	6	*Pseudomonas aeruginosa*
5	*Streptococcus pyogenes*	6	*Oligella ureolytica*
5	*Streptococcus equi*	6	*Ochrobactrum anthropi*
5	*Pseudomonas aeruginosa*	6	*Aggregatibacter aphrophilus*
5	*Ochrobactrum anthropi*	5	*Streptococcus equi*
5	*Haloplasma contractile*	5	*Enterococcus faecalis*
5	*Enterococcus faecium*	5	*Enterococcus casseliflavus*
5	*Corynebacterium simulans*	4	*Staphylococcus aureus*
5	*Bacteroides ovatus*	4	*Shigella boydii*
5	*Aggregatibacter aphrophilus*	4	*Escherichia coli*
4	*Staphylococcus aureus*	4	*Enterococcus faecium*
4	*Shigella boydii*	4	*Enterobacteriaceae bacterium*
4	*Brevibacillus agri*	4	*Eikenella corrodens*
3	*Paenibacillus kribbensis*	3	*Enterococcus gallinarum*
3	*Haemophilus aegyptius*	2	*Streptococcus salivarius*
3	*Enterococcus camelliae*	2	*Staphylococcus epidermidis*
3	*Clostridium perfringens*	2	*Shigella flexneri*
2	*Streptococcus thermophilus*	2	*Paenibacillus kribbensis*
2	*Shigella flexneri*	2	*Elizabethkingia meningoseptica*
2	*Oceanobacillus indicireducens*	2	*Clostridium perfringens*
2	*Enterococcus faecalis*	1	*Stenotrophomonas maltophilia*
2	*Elizabethkingia meningoseptica*	1	*Paenibacillus peoriae*
2	*Eikenella corrodens*	1	*Ochrobactrum tritici*
2	*Bacillus amyloliquefaciens*	1	*Enterobacter cloacae*

1	*Ureibacillus terrenus*	24350	Total Reads
1	*Turicibacter sanguinis*		
1	*Streptococcus mitis*		
1	*Stenotrophomonas maltophilia*		
1	*Staphylococcus epidermidis*		
1	*Shigella dysenteriae*		
1	*Proteus hauseri*		
1	*Paenibacillus ehimensis*		
1	*Listeria fleischmannii*		
1	*Haemophilus influenzae*		
1	*Citrobacter koseri*		
1	*Bacillus siralis*		
1	*Bacillus chagannorensis*		
1	*Aneurinibacillus aneurinilyticus*		
16175	Total Reads		

Organism sequenced: *Oligella ureolytica* ATCC 43534

blastn number of reads matched	blastn organism matched	SequenceMatch number of reads matched	SequenceMatch organism matched
63147	*Oligella ureolytica*	65967	*Oligella ureolytica*
22	*Klebsiella oxytoca*	21	*Klebsiella oxytoca*
12	*Neisseria gonorrhoeae*	13	*Neisseria gonorrhoeae*
12	*Eikenella corrodens*	12	*Eikenella corrodens*
8	*Streptococcus pneumoniae*	10	*Streptococcus pneumoniae*
8	*Shigella sonnei*	7	*Staphylococcus sciuri*
7	*Staphylococcus sciuri*	7	*Aggregatibacter aphrophilus*
6	*Proteus mirabilis*	6	*Proteus mirabilis*
5	*Listeria monocytogenes*	6	*Listeria monocytogenes*
4	*Proteus vulgaris*	6	*Bacteroides ovatus*
4	*Aggregatibacter aphrophilus*	5	*Stenotrophomonas maltophilia*
3	*Streptococcus thermophilus*	5	*Proteus vulgaris*
3	*Stenotrophomonas maltophilia*	5	*Enterobacteriaceae bacterium*
3	*Ochrobactrum anthropi*	4	*Ochrobactrum anthropi*
2	*Streptococcus pyogenes*	3	*Streptococcus salivarius*
2	*Shigella boydii*	3	*Streptococcus pyogenes*
2	*Pseudomonas aeruginosa*	3	*Kocuria rosea*
2	*Escherichia fergusonii*	3	*Corynebacterium striatum*
2	*Elizabethkingia meningoseptica*	2	*Shigella sonnei*
2	*Corynebacterium simulans*	2	*Shigella boydii*
2	*Clostridium septicum*	2	*Pseudomonas aeruginosa*
2	*Bacteroides ovatus*	2	*Haemophilus influenzae*
1	*Staphylococcus aureus*	2	*Escherichia coli*
1	*Shigella dysenteriae*	2	*Enterococcus faecium*
1	*Paenibacillus polymyxa*	2	*Enterobacter hormaechei*
1	*Kocuria kristinae*	2	*Elizabethkingia meningoseptica*
1	*Haemophilus influenzae*	2	*Clostridium septicum*

1	*Enterococcus saccharolyticus*	2	*Citrobacter freundii*
1	*Enterococcus faecium*	1	*Staphylococcus aureus*
1	*Clostridium perfringens*	1	*Paenibacillus polymyxa*
63268	Total Reads	1	*Enterococcus saccharolyticus*
		1	*Enterococcus faecalis*
		1	*Enterococcus durans*
		1	*[Clostridium] sordellii*
		1	*Clostridium perfringens*
		66113	Total Reads

Organism sequenced: *Pseudomonas aeruginosa* ATCC 27853			
blastn number of reads matched	blastn organism matched	SequenceMatch number of reads matched	SequenceMatch organism matched
63332	*Pseudomonas aeruginosa*	71376	*Pseudomonas aeruginosa*
39	*Klebsiella oxytoca*	37	*Klebsiella oxytoca*
30	*Streptococcus lutetiensis*	35	*Klebsiella pneumoniae*
24	*Streptococcus equinus*	32	*Streptococcus equinus*
18	*Shigella sonnei*	31	*Streptococcus lutetiensis*
16	*Listeria monocytogenes*	25	*Pseudomonas thermaerum*
11	*Neisseria gonorrhoeae*	25	*Listeria monocytogenes*
9	*Stenotrophomonas maltophilia*	12	*Neisseria gonorrhoeae*
9	*Proteus mirabilis*	11	*Proteus vulgaris*
9	*Bacteroides ovatus*	11	*Proteus mirabilis*
8	*Staphylococcus aureus*	11	*Bacteroides ovatus*
7	*Oligella ureolytica*	10	*Stenotrophomonas maltophilia*
7	*Clostridium septicum*	10	*Haemophilus influenzae*
7	*Aggregatibacter aphrophilus*	9	*Aggregatibacter aphrophilus*
6	*Streptococcus pneumoniae*	8	*Staphylococcus sciuri*
5	*Staphylococcus sciuri*	8	*Staphylococcus aureus*
5	*Enterococcus saccharolyticus*	8	*Escherichia coli*
5	*Enterococcus casseliflavus*	8	*Enterococcus saccharolyticus*
4	*Streptococcus pyogenes*	8	*Enterobacteriaceae bacterium*
4	*Proteus vulgaris*	8	*Eikenella corrodens*
4	*Ochrobactrum anthropi*	8	*[Clostridium] sordellii*
4	*Eikenella corrodens*	7	*Streptococcus pneumoniae*
3	*Streptococcus equi*	7	*Shigella sonnei*
3	*Shigella dysenteriae*	7	*Paenibacillus polymyxa*
3	*Shigella boydii*	7	*Oligella ureolytica*
3	*Elizabethkingia meningoseptica*	7	*Ochrobactrum anthropi*
3	*Clostridium perfringens*	7	*Clostridium septicum*
2	*Streptococcus thermophilus*	6	*Streptococcus pyogenes*
2	*Paenibacillus polymyxa*	5	*Streptococcus infantarius*
2	*[Clostridium] sordellii*	4	*Streptococcus equi*
1	*Streptococcus infantarius*	4	*Enterococcus casseliflavus*

1	*Staphylococcus saprophyticus*	4	*Clostridium perfringens*
1	*Staphylococcus epidermidis*	3	*Streptococcus salivarius*
1	*Shigella flexneri*	3	*Shigella flexneri*
1	*Kocuria kristinae*	3	*Shigella boydii*
1	*Haemophilus influenzae*	3	*Elizabethkingia meningoseptica*
1	*Haemophilus aegyptius*	3	*Corynebacterium striatum*
1	*Corynebacterium simulans*	2	*Enterococcus gallinarum*
1	*Citrobacter freundii*	2	*Citrobacter freundii*
63593	Total Reads	1	*Staphylococcus saprophyticus*
		1	*Staphylococcus epidermidis*
		1	*Ochrobactrum tritici*
		1	*Kocuria rosea*
		71779	Total Reads

Organism sequenced: *Shigella sonnei* ATCC 25931

blastn number of reads matched	blastn organism matched	SequenceMatch number of reads matched	SequenceMatch organism matched
65917	*Shigella sonnei*	43171	*Shigella sonnei*
12151	*Shigella boydii*	30966	*Enterobacteriaceae bacterium*
10297	*Shigella flexneri*	11344	*Enterobacter hormaechei*
5489	*Escherichia fergusonii*	7986	*Shigella flexneri*
4563	*Citrobacter koseri*	7574	*Escherichia coli*
391	*Escherichia coli*	6343	*Shigella boydii*
58	*Citrobacter freundii*	884	*Enterobacter ludwigii*
57	*Escherichia albertii*	419	*Enterobacter cancerogenus*
55	*Klebsiella oxytoca*	150	*Enterobacter cloacae*
44	*Enterobacter cancerogenus*	95	*Shigella dysenteriae*
24	*Enterobacter mori*	53	*Klebsiella oxytoca*
20	*Shigella dysenteriae*	26	*Listeria monocytogenes*
16	*Proteus vulgaris*	21	*Proteus vulgaris*
16	*Listeria monocytogenes*	17	*Neisseria gonorrhoeae*
15	*Neisseria gonorrhoeae*	15	*Streptococcus pneumoniae*
14	*Salmonella enterica*	15	*Aggregatibacter aphrophilus*
13	*Aggregatibacter aphrophilus*	14	*Proteus mirabilis*
12	*Streptococcus pneumoniae*	13	*Enterococcus saccharolyticus*
10	*Staphylococcus sciuri*	11	*Staphylococcus sciuri*
10	*Proteus mirabilis*	11	*Haemophilus influenzae*
10	*Enterococcus saccharolyticus*	11	*Bacteroides ovatus*
10	*Bacteroides ovatus*	9	*Stenotrophomonas maltophilia*
9	*Mangrovibacter plantisponsor*	8	*Pseudomonas aeruginosa*
8	*Citrobacter farmeri*	8	*Eikenella corrodens*
7	*Salmonella subterranea*	8	*Citrobacter farmeri*
6	*Stenotrophomonas maltophilia*	7	*Ochrobactrum anthropi*
6	*Oligella ureolytica*	7	*Escherichia hermannii*
6	*Eikenella corrodens*	7	*Enterobacter asburiae*

5	*Pseudomonas aeruginosa*	6	*Oligella ureolytica*
5	*Haemophilus influenzae*	6	*Escherichia albertii*
5	*Haemophilus aegyptius*	6	*Enterococcus faecium*
5	*Corynebacterium simulans*	5	*Escherichia fergusonii*
4	*Ochrobactrum anthropi*	5	*Corynebacterium striatum*
4	*Kosakonia cowanii*	4	*Salmonella bongori*
3	*Streptococcus pyogenes*	4	*Enterobacter massiliensis*
3	*Streptococcus equi*	4	*[Clostridium] sordellii*
3	*Enterococcus faecium*	4	*Clostridium perfringens*
3	*Enterococcus casseliflavus*	4	*Citrobacter koseri*
3	*Enterococcus camelliae*	3	*Streptococcus pyogenes*
3	*Clostridium septicum*	3	*Streptococcus equi*
3	*Clostridium perfringens*	3	*Proteus hauseri*
2	*Streptococcus thermophilus*	3	*Paenibacillus polymyxa*
2	*Staphylococcus epidermidis*	3	*Clostridium septicum*
2	*Staphylococcus aureus*	3	*Citrobacter freundii*
2	*Proteus hauseri*	2	*Streptococcus salivarius*
2	*Paenibacillus polymyxa*	2	*Staphylococcus epidermidis*
2	*Kocuria kristinae*	2	*Staphylococcus aureus*
2	*Enterobacter hormaechei*	2	*Kocuria rosea*
1	*Staphylococcus saprophyticus*	2	*Escherichia/Shigella flexneri*
1	*Raoultella planticola*	2	*Enterococcus gallinarum*
1	*Listeria innocua*	2	*Enterococcus faecalis*
1	*Erwinia mallotivora*	2	*Enterococcus casseliflavus*
1	*Enterococcus faecalis*	1	*Staphylococcus saprophyticus*
1	*Cronobacter helveticus*	1	*Proteus penneri*
1	*[Clostridium] sordellii*	1	*Grimontella senegalensis*
99304	Total Reads	1	*Elizabethkingia meningoseptica*
		1	*Citrobacter braakii*
		109280	Total Reads
Organism sequenced: *Ochrobactrum anthropi* ATCC BAA 749			
blastn number of reads matched	blastn organism matched	SequenceMatch number of reads matched	SequenceMatch organism matched
65411	*Ochrobactrum anthropi*	87778	*Ochrobactrum anthropi*
29	*Klebsiella oxytoca*	2395	*Ochrobactrum cytisi*
17	*Shigella sonnei*	1252	*Ochrobactrum tritici*
13	*Listeria monocytogenes*	243	*Ochrobactrum lupini*
8	*Enterococcus casseliflavus*	32	*Klebsiella oxytoca*
6	*Streptococcus pneumoniae*	22	*Pedobacter kribbensis*
6	*Proteus vulgaris*	17	*Listeria monocytogenes*
6	*Neisseria gonorrhoeae*	9	*Proteus vulgaris*
6	*Enterococcus saccharolyticus*	9	*Eikenella corrodens*
6	*Eikenella corrodens*	8	*Neisseria gonorrhoeae*
5	*Shigella boydii*	8	*Enterococcus casseliflavus*

5	*Proteus mirabilis*	8	*Bacteroides ovatus*
5	*Oligella ureolytica*	7	*Shigella sonnei*
5	*[Clostridium] sordellii*	7	*Paenibacillus polymyxa*
5	*Aggregatibacter aphrophilus*	7	*Escherichia coli*
4	*Streptococcus thermophilus*	7	*Enterobacteriaceae bacterium*
4	*Staphylococcus sciuri*	6	*Streptococcus pneumoniae*
4	*Staphylococcus epidermidis*	6	*Enterococcus saccharolyticus*
4	*Pseudomonas aeruginosa*	6	*[Clostridium] sordellii*
4	*Paenibacillus polymyxa*	6	*Aggregatibacter aphrophilus*
4	*Bacteroides ovatus*	5	*Staphylococcus sciuri*
3	*Staphylococcus aureus*	5	*Proteus mirabilis*
3	*Escherichia fergusonii*	5	*Oligella ureolytica*
2	*Streptococcus pyogenes*	5	*Enterobacter hormaechei*
2	*Streptococcus equi*	4	*Streptococcus salivarius*
2	*Stenotrophomonas maltophilia*	4	*Staphylococcus epidermidis*
2	*Staphylococcus saprophyticus*	4	*Pseudomonas aeruginosa*
2	*Shigella dysenteriae*	3	*Streptococcus pyogenes*
2	*Proteus hauseri*	3	*Streptococcus equi*
2	*Paenibacillus jamilae*	3	*Staphylococcus aureus*
2	*Clostridium septicum*	3	*Shigella boydii*
1	*Shigella flexneri*	3	*Haemophilus influenzae*
1	*Ochrobactrum tritici*	3	*Clostridium perfringens*
1	*Haemophilus influenzae*	2	*Stenotrophomonas maltophilia*
1	*Enterococcus faecium*	2	*Staphylococcus saprophyticus*
1	*Enterococcus faecalis*	2	*Proteus hauseri*
1	*Elizabethkingia meningoseptica*	2	*Enterococcus gallinarum*
1	*Citrobacter koseri*	2	*Enterococcus faecalis*
1	*Bartonella clarridgeiae*	2	*Clostridium septicum*
1	*Bacillus composti*	2	*Citrobacter freundii*
1	*Acidovorax ebreus*	2	*Brucella ceti*
65589	Total Reads	1	*Shigella flexneri*
		1	*Neoasaia chiangmaiensis*
		1	*Kozakia baliensis*
		1	*Enterococcus faecium*
		1	*Enterobacter ludwigii*
		1	*Elizabethkingia meningoseptica*
		1	*Acidovorax ebreus*
		91906	Total Reads
Organism sequenced: *Listeria monocytogenes* ATCC BAA 751			
blastn number of reads matched	blastn organism matched	SequenceMatch number of reads matched	SequenceMatch organism matched
27641	*Listeria monocytogenes*	44878	*Listeria monocytogenes*
3851	*Enterococcus camelliae*	31	*Klebsiella oxytoca*
159	*Enterococcus sulfureus*	26	*Ochrobactrum anthropi*

51	*Listeria innocua*	15	*Clostridium septicum*
30	*Klebsiella oxytoca*	14	*Enterococcus saccharolyticus*
23	*Ochrobactrum anthropi*	12	*Staphylococcus aureus*
16	*Shigella sonnei*	12	*Neisseria gonorrhoeae*
13	*Clostridium septicum*	11	*Streptococcus pneumoniae*
11	*Staphylococcus aureus*	11	*Paenibacillus polymyxa*
10	*Neisseria gonorrhoeae*	11	*[Clostridium] sordellii*
10	*Enterococcus saccharolyticus*	10	*Staphylococcus sciuri*
9	*Enterococcus casseliflavus*	9	*Streptococcus pyogenes*
8	*Streptococcus pyogenes*	9	*Corynebacterium striatum*
8	*Streptococcus pneumoniae*	9	*Clostridium perfringens*
8	*Clostridium perfringens*	9	*Bacteroides ovatus*
7	*Streptococcus thermophilus*	8	*Proteus vulgaris*
6	*Pseudomonas aeruginosa*	8	*Enterococcus faecalis*
6	*Enterococcus faecium*	8	*Enterobacteriaceae bacterium*
6	*[Clostridium] sordellii*	7	*Streptococcus salivarius*
5	*Staphylococcus sciuri*	7	*Pseudomonas aeruginosa*
5	*Proteus vulgaris*	7	*Haemophilus influenzae*
5	*Paenibacillus polymyxa*	7	*Enterococcus faecium*
5	*Enterococcus faecalis*	7	*Eikenella corrodens*
5	*Corynebacterium simulans*	5	*Shigella sonnei*
5	*Bacteroides ovatus*	5	*Enterococcus casseliflavus*
4	*Stenotrophomonas maltophilia*	4	*Stenotrophomonas maltophilia*
4	*Proteus mirabilis*	4	*Staphylococcus epidermidis*
4	*Oligella ureolytica*	4	*Proteus mirabilis*
4	*Eikenella corrodens*	4	*Oligella ureolytica*
3	*Shigella flexneri*	4	*Listeria innocua*
3	*Elizabethkingia meningoseptica*	4	*Enterococcus gallinarum*
2	*Staphylococcus saprophyticus*	3	*Shigella flexneri*
2	*Staphylococcus epidermidis*	3	*Ochrobactrum cytisi*
2	*Staphylococcus capitis*	3	*Enterobacter hormaechei*
2	*Shigella boydii*	3	*Elizabethkingia meningoseptica*
2	*Lysinibacillus xylanilyticus*	3	*Citrobacter freundii*
1	*Streptococcus equi*	3	*Aggregatibacter aphrophilus*
1	*Paenibacillus jamilae*	2	*Shigella boydii*
1	*Haemophilus influenzae*	1	*Streptococcus equi*
1	*Haemophilus aegyptius*	1	*Staphylococcus xylosus*
1	*Enterococcus thailandicus*	1	*Staphylococcus saprophyticus*
1	*Enterococcus italicus*	1	*Paenibacillus jamilae*
1	*Clostridium intestinale*	1	*Listeria welshimeri*
1	*Aggregatibacter aphrophilus*	1	*Kocuria rosea*
31943	Total Reads	1	*Enterobacter ludwigii*
		45187	Total Reads
Organism sequenced: *Proteus vulgaris* ATCC 6380			

blastn number of reads matched	blastn organism matched	SequenceMatch number of reads matched	SequenceMatch organism matched
57307	*Proteus vulgaris*	66893	*Proteus vulgaris*
7453	*Proteus hauseri*	7681	*Proteus mirabilis*
238	*Proteus penneri*	1940	*Proteus hauseri*
24	*Proteus mirabilis*	16	*Klebsiella oxytoca*
12	*Klebsiella oxytoca*	13	*Listeria monocytogenes*
9	*Bacteroides ovatus*	11	*Bacteroides ovatus*
8	*Staphylococcus sciuri*	8	*Staphylococcus sciuri*
7	*Shigella sonnei*	7	*Oligella ureolytica*
7	*Listeria monocytogenes*	7	*[Clostridium] sordellii*
5	*Neisseria gonorrhoeae*	5	*Neisseria gonorrhoeae*
4	*Streptococcus thermophilus*	5	*Escherichia coli*
3	*Pseudomonas aeruginosa*	5	*Enterobacteriaceae bacterium*
3	*Oligella ureolytica*	4	*Streptococcus salivarius*
3	*Ochrobactrum anthropi*	4	*Haemophilus influenzae*
3	*Haemophilus influenzae*	4	*Eikenella corrodens*
3	*Corynebacterium simulans*	4	*Clostridium septicum*
3	*[Clostridium] sordellii*	3	*Stenotrophomonas maltophilia*
3	*Clostridium septicum*	3	*Shigella sonnei*
3	*Aggregatibacter aphrophilus*	3	*Pseudomonas aeruginosa*
2	*Streptococcus equi*	3	*Proteus penneri*
2	*Stenotrophomonas maltophilia*	3	*Ochrobactrum anthropi*
2	*Shigella flexneri*	3	*Enterococcus saccharolyticus*
2	*Shigella dysenteriae*	3	*Enterococcus faecalis*
2	*Enterococcus saccharolyticus*	3	*Elizabethkingia meningoseptica*
2	*Enterococcus casseliflavus*	3	*Corynebacterium striatum*
2	*Elizabethkingia meningoseptica*	3	*Aggregatibacter aphrophilus*
2	*Eikenella corrodens*	2	*Streptococcus equi*
2	*Clostridium perfringens*	2	*Staphylococcus epidermidis*
1	*Streptococcus pneumoniae*	2	*Staphylococcus aureus*
1	*Staphylococcus epidermidis*	2	*Enterobacter hormaechei*
1	*Staphylococcus aureus*	2	*Clostridium perfringens*
1	*Shigella boydii*	1	*Streptococcus pneumoniae*
1	*Paenibacillus polymyxa*	1	*Shigella boydii*
1	*Escherichia fergusonii*	1	*Serratia odorifera*
1	*Escherichia coli*	1	*Paenibacillus polymyxa*
1	*Enterococcus faecium*	1	*Enterococcus gallinarum*
1	*Enterococcus faecalis*	1	*Enterococcus faecium*
1	*Citrobacter koseri*	1	*Enterococcus casseliflavus*
65126	Total Reads	1	*Enterobacter ludwigii*
		1	*Citrobacter freundii*
		76656	Total Reads
Organism sequenced: *Bacteroides ovatus* ATCC 1296			

blastn number of reads matched	blastn organism matched	SequenceMatch number of reads matched	SequenceMatch organism matched
35982	*Bacteroides ovatus*	54389	*Bacteroides ovatus*
36	*Klebsiella oxytoca*	41	*Klebsiella oxytoca*
24	*Eikenella corrodens*	31	*Eikenella corrodens*
18	*Shigella sonnei*	21	*Listeria monocytogenes*
14	*Neisseria gonorrhoeae*	21	*[Clostridium] sordellii*
14	*Listeria monocytogenes*	19	*Staphylococcus sciuri*
12	*Enterococcus faecalis*	15	*Enterococcus faecalis*
11	*Streptococcus pneumoniae*	14	*Ochrobactrum anthropi*
10	*Staphylococcus sciuri*	14	*Neisseria gonorrhoeae*
10	*Aggregatibacter aphrophilus*	13	*Streptococcus pneumoniae*
9	*Stenotrophomonas maltophilia*	12	*Stenotrophomonas maltophilia*
9	*Enterococcus saccharolyticus*	12	*Enterococcus saccharolyticus*
9	*Clostridium septicum*	11	*Haemophilus influenzae*
8	*Ochrobactrum anthropi*	11	*Aggregatibacter aphrophilus*
7	*Staphylococcus epidermidis*	10	*Enterobacteriaceae bacterium*
7	*Pseudomonas aeruginosa*	9	*Shigella sonnei*
7	*Elizabethkingia meningoseptica*	9	*Pseudomonas aeruginosa*
6	*Proteus vulgaris*	9	*Proteus vulgaris*
6	*Oligella ureolytica*	9	*Clostridium septicum*
6	*[Clostridium] sordellii*	8	*Staphylococcus epidermidis*
5	*Staphylococcus saprophyticus*	8	*Oligella ureolytica*
5	*Staphylococcus aureus*	8	*Escherichia coli*
5	*Proteus mirabilis*	8	*Elizabethkingia meningoseptica*
4	*Streptococcus pyogenes*	7	*Staphylococcus aureus*
4	*Shigella dysenteriae*	6	*Proteus mirabilis*
4	*Shigella boydii*	6	*Paenibacillus polymyxa*
4	*Haemophilus influenzae*	5	*Staphylococcus saprophyticus*
4	*Corynebacterium simulans*	5	*Sphingomonas bosoensis*
3	*Haemophilus aegyptius*	5	*Shigella boydii*
3	*Escherichia coli*	5	*Corynebacterium striatum*
3	*Enterococcus faecium*	4	*Streptococcus pyogenes*
3	*Clostridium perfringens*	4	*Enterococcus faecium*
2	*Streptococcus thermophilus*	4	*Clostridium perfringens*
2	*Shigella flexneri*	2	*Streptococcus salivarius*
2	*Paenibacillus polymyxa*	2	*Streptococcus equi*
2	*Gracilibacillus dipsosauri*	2	*Shigella flexneri*
2	*Citrobacter koseri*	1	*Enterococcus gallinarum*
1	*Streptococcus equi*	1	*Citrobacter freundii*
1	*Enterococcus casseliflavus*	54761	Total Reads
36264	Total Reads		

Organism sequenced: *Clostridium sordellii* ATCC 9714

blastn number of reads matched	blastn organism matched	SequenceMatch number of reads matched	SequenceMatch organism matched
30206	*[Clostridium] sordellii*	65976	*[Clostridium] sordellii*
22	*Eikenella corrodens*	439	*Clostridium sordellii*
14	*Enterococcus saccharolyticus*	25	*Eikenella corrodens*
12	*Bacteroides ovatus*	18	*Listeria monocytogenes*
11	*Shigella sonnei*	17	*Bacteroides ovatus*
11	*Klebsiella oxytoca*	16	*Enterococcus saccharolyticus*
10	*Staphylococcus sciuri*	11	*Klebsiella oxytoca*
10	*Listeria monocytogenes*	10	*Staphylococcus sciuri*
8	*Ochrobactrum anthropi*	9	*Ochrobactrum anthropi*
6	*Enterococcus casseliflavus*	7	*Streptococcus pneumoniae*
5	*Streptococcus pneumoniae*	7	*Enterobacteriaceae bacterium*
5	*Clostridium perfringens*	6	*Shigella sonnei*
4	*Proteus mirabilis*	5	*Proteus vulgaris*
4	*Paenibacillus polymyxa*	5	*Escherichia coli*
3	*Staphylococcus aureus*	4	*Staphylococcus aureus*
3	*Proteus vulgaris*	4	*Proteus mirabilis*
3	*Enterococcus faecium*	4	*Paenibacillus polymyxa*
3	*Aggregatibacter aphrophilus*	4	*Enterococcus gallinarum*
2	*Streptococcus pyogenes*	4	*Enterococcus casseliflavus*
2	*Staphylococcus saprophyticus*	4	*Clostridium perfringens*
2	*Shigella dysenteriae*	4	*Aggregatibacter aphrophilus*
2	*Oligella ureolytica*	3	*Streptococcus pyogenes*
2	*Neisseria gonorrhoeae*	3	*Staphylococcus saprophyticus*
2	*Enterococcus faecalis*	3	*Haemophilus influenzae*
2	*Corynebacterium simulans*	3	*Enterococcus faecium*
2	*Clostridium septicum*	3	*Corynebacterium striatum*
1	*Streptococcus thermophilus*	2	*Streptococcus salivarius*
1	*Streptococcus equi*	2	*Streptococcus equi*
1	*Stenotrophomonas maltophilia*	2	*Stenotrophomonas maltophilia*
1	*Staphylococcus epidermidis*	2	*Pseudomonas aeruginosa*
1	*Shigella flexneri*	2	*Paenibacillus jamilae*
1	*Shigella boydii*	2	*Oligella ureolytica*
1	*Pseudomonas aeruginosa*	2	*Neisseria gonorrhoeae*
1	*Paenibacillus jamilae*	2	*Enterococcus faecalis*
1	*Novosphingobium rosa*	2	*Clostridium septicum*
1	*Escherichia coli*	1	*Staphylococcus epidermidis*
1	*Enterococcus camelliae*	1	*Sphingomonas rosa*
1	*Elizabethkingia meningoseptica*	1	*Shigella flexneri*
1	*Citrobacter koseri*	1	*Ochrobactrum tritici*
30369	Total Reads	1	*Eubacterium tenue*
		1	*Elizabethkingia meningoseptica*
		1	*Clostridiaceae bacterium*

		66619	Total Reads
Organism sequenced: *Eikenella corrodens* BAA 1152			
blastn number of reads matched	blastn organism matched	SequenceMatch number of reads matched	SequenceMatch organism matched
84673	*Eikenella corrodens*	100224	*Eikenella corrodens*
23	*Klebsiella oxytoca*	24	*Klebsiella oxytoca*
14	*Oligella ureolytica*	17	*Listeria monocytogenes*
13	*Clostridium perfringens*	14	*Oligella ureolytica*
10	*Neisseria gonorrhoeae*	14	*Clostridium perfringens*
10	*Listeria monocytogenes*	13	*Neisseria gonorrhoeae*
8	*Proteus mirabilis*	9	*Proteus mirabilis*
7	*Streptococcus pneumoniae*	7	*Streptococcus pneumoniae*
7	*Stenotrophomonas maltophilia*	7	*Stenotrophomonas maltophilia*
6	*Shigella sonnei*	7	*Staphylococcus epidermidis*
5	*Staphylococcus epidermidis*	7	*Shigella sonnei*
5	*Pseudomonas aeruginosa*	7	*Pseudomonas aeruginosa*
4	*Streptococcus thermophilus*	6	*Escherichia coli*
4	*Ochrobactrum anthropi*	5	*Paenibacillus polymyxa*
4	*Enterococcus casseliflavus*	5	*Haemophilus influenzae*
3	*Streptococcus pyogenes*	4	*Streptococcus salivarius*
3	*Staphylococcus aureus*	4	*Streptococcus pyogenes*
3	*Shigella dysenteriae*	4	*Ochrobactrum anthropi*
3	*Haemophilus influenzae*	4	*Enterococcus saccharolyticus*
3	*Enterococcus saccharolyticus*	4	*Aggregatibacter aphrophilus*
3	*Enterococcus camelliae*	3	*Streptococcus equi*
3	*Elizabethkingia meningoseptica*	3	*Staphylococcus aureus*
2	*Streptococcus equi*	3	*Enterococcus gallinarum*
2	*Staphylococcus saprophyticus*	3	*Enterococcus casseliflavus*
2	*Shigella flexneri*	3	*Enterobacteriaceae bacterium*
2	*Shigella boydii*	3	*Enterobacter hormaechei*
2	*Proteus vulgaris*	3	*Elizabethkingia meningoseptica*
2	*Proteus hauseri*	3	*Corynebacterium striatum*
2	*Enterococcus faecium*	3	*Citrobacter freundii*
2	*Citrobacter koseri*	3	*Bacteroides ovatus*
2	*Bacteroides ovatus*	2	*Staphylococcus sciuri*
2	*Aggregatibacter aphrophilus*	2	*Staphylococcus saprophyticus*
1	*Staphylococcus sciuri*	2	*Proteus vulgaris*
1	*Paenibacillus polymyxa*	2	*Enterococcus faecium*
1	*Paenibacillus jamilae*	2	*[Clostridium] sordellii*
1	*Haemophilus aegyptius*	2	*Clostridium septicum*
1	*Escherichia fergusonii*	2	*Burkholderiales bacterium*
1	*Enterococcus faecalis*	1	*Shigella flexneri*
1	*Enterobacter hormaechei*	1	*Shigella boydii*
1	*Corynebacterium simulans*	1	*Proteus hauseri*

1	*[Clostridium] sordellii*	1	*Neisseria meningitidis*
1	*Clostridium septicum*	1	*Kocuria rosea*
1	*Bacillus fordii*	1	*Enterococcus faecalis*
84845	Total Reads	100436	Total Reads
Organism sequenced: *Clostridium perfringens* ATCC 13124			
blastn number of reads matched	blastn organism matched	SequenceMatch number of reads matched	SequenceMatch organism matched
39999	*Clostridium perfringens*	42606	*Clostridium perfringens*
708	*Bacillus composti*	1422	*Clostridiaceae bacterium*
38	*Bacillus fordii*	34	*Listeria monocytogenes*
27	*Klebsiella oxytoca*	30	*Klebsiella oxytoca*
20	*Listeria monocytogenes*	22	*Enterococcus saccharolyticus*
19	*Neisseria gonorrhoeae*	21	*Bacteroides ovatus*
19	*Enterococcus saccharolyticus*	20	*Neisseria gonorrhoeae*
15	*Staphylococcus sciuri*	17	*Staphylococcus sciuri*
14	*Clostridium septicum*	16	*Clostridium septicum*
13	*Streptococcus pneumoniae*	15	*Streptococcus pneumoniae*
11	*Bacteroides ovatus*	14	*Ochrobactrum anthropi*
10	*Ochrobactrum anthropi*	13	*Paenibacillus polymyxa*
9	*Streptococcus pyogenes*	13	*[Clostridium] sordellii*
8	*Stenotrophomonas maltophilia*	11	*Proteus mirabilis*
8	*Paenibacillus polymyxa*	10	*Streptococcus pyogenes*
7	*Shigella sonnei*	9	*Stenotrophomonas maltophilia*
7	*Pseudomonas aeruginosa*	9	*Shigella sonnei*
7	*Proteus mirabilis*	9	*Proteus vulgaris*
7	*Aggregatibacter aphrophilus*	9	*Clostridiales bacterium*
6	*Staphylococcus epidermidis*	9	*Aggregatibacter aphrophilus*
6	*Oligella ureolytica*	8	*Haemophilus influenzae*
5	*Staphylococcus aureus*	7	*Staphylococcus epidermidis*
5	*Shigella flexneri*	7	*Pseudomonas aeruginosa*
4	*Proteus vulgaris*	7	*Enterococcus faecium*
4	*Enterococcus casseliflavus*	7	*Enterococcus casseliflavus*
4	*Elizabethkingia meningoseptica*	7	*Corynebacterium striatum*
4	*Eikenella corrodens*	6	*Staphylococcus aureus*
4	*Corynebacterium simulans*	6	*Oligella ureolytica*
3	*Streptococcus thermophilus*	5	*Escherichia coli*
3	*Shigella dysenteriae*	5	*Eikenella corrodens*
3	*Planococcus plakortidis*	4	*Enterococcus faecalis*
3	*Paenibacillus jamilae*	4	*Enterobacteriaceae bacterium*
3	*Haemophilus influenzae*	4	*Elizabethkingia meningoseptica*
3	*Enterococcus faecium*	3	*Enterobacter hormaechei*
3	*Enterococcus faecalis*	2	*Streptococcus salivarius*
3	*[Clostridium] sordellii*	2	*Staphylococcus saprophyticus*
2	*Staphylococcus saprophyticus*	1	*Shigella flexneri*
2	*Sporosarcina saromensis*	1	*Ochrobactrum cytisi*

2	*Oceanobacillus chironomi*	1	*Enterococcus hirae*
2	*Haemophilus aegyptius*	1	*Enterococcus gallinarum*
2	*Enterococcus camelliae*	1	*Citrobacter freundii*
2	*Bacillus farraginis*	44398	Total Reads
1	*Virgibacillus zhanjiangensis*		
1	*Streptococcus equi*		
1	*Streptococcus dysgalactiae*		
1	*Shigella boydii*		
1	*Proteus hauseri*		
1	*Escherichia fergusonii*		
1	*Enterococcus canis*		
1	*Citrobacter koseri*		
1	*Bacillus vietnamensis*		
1	*Bacillus thermophilus*		
1	*Bacillus subtilis*		
1	*Bacillus chagannorensis*		
1	*Aneurinibacillus migulanus*		
41037	Total Reads		

Organism sequenced: *Clostridium septicum* ATCC 12464

blastn number of reads matched	blastn organism matched	SequenceMatch number of reads matched	SequenceMatch organism matched
78874	*Clostridium septicum*	82577	*Clostridium septicum*
493	*Bacillus siralis*	67	*Listeria monocytogenes*
492	*Lysinibacillus xylanilyticus*	38	*Klebsiella oxytoca*
40	*Listeria monocytogenes*	34	*Enterococcus saccharolyticus*
38	*Klebsiella oxytoca*	25	*Staphylococcus sciuri*
27	*Enterococcus saccharolyticus*	25	*Neisseria gonorrhoeae*
24	*Neisseria gonorrhoeae*	24	*Streptococcus pneumoniae*
23	*Shigella sonnei*	19	*Proteus mirabilis*
21	*Staphylococcus sciuri*	18	*[Clostridium] sordellii*
21	*[Flavobacterium] thermophilum*	17	*Paenibacillus polymyxa*
18	*Streptococcus pneumoniae*	16	*Shigella sonnei*
16	*Enterococcus casseliflavus*	15	*Pseudomonas aeruginosa*
15	*Pseudomonas aeruginosa*	15	*Proteus vulgaris*
15	*Bacillus luciferensis*	15	*Aggregatibacter aphrophilus*
14	*Proteus vulgaris*	14	*Escherichia coli*
14	*Paenibacillus wooponensis*	13	*Staphylococcus aureus*
13	*Clostridium perfringens*	13	*Clostridium perfringens*
13	*Anoxybacillus voinovskiensis*	13	*Bacteroides ovatus*
12	*Proteus mirabilis*	12	*Staphylococcus epidermidis*
11	*Bacteroides ovatus*	12	*Ochrobactrum anthropi*
11	*Aggregatibacter aphrophilus*	12	*Enterococcus casseliflavus*
10	*Staphylococcus epidermidis*	12	*Corynebacterium striatum*
9	*Streptococcus pyogenes*	11	*Streptococcus pyogenes*

9	*Elizabethkingia meningoseptica*	10	*Elizabethkingia meningoseptica*
9	*Corynebacterium simulans*	8	*Enterobacteriaceae bacterium*
9	*[Clostridium] sordellii*	8	*Eikenella corrodens*
7	*Staphylococcus aureus*	7	*Pseudomonas chlororaphis*
7	*Paenibacillus polymyxa*	7	*Enterococcus faecalis*
7	*Eikenella corrodens*	6	*Haemophilus influenzae*
6	*Shigella dysenteriae*	6	*Enterococcus faecium*
6	*Pseudomonas cedrina*	4	*Stenotrophomonas maltophilia*
6	*Bacillus tianmuensis*	4	*Oligella ureolytica*
6	*Bacillus halodurans*	4	*Citrobacter freundii*
5	*Shigella boydii*	3	*Streptococcus salivarius*
5	*Ochrobactrum anthropi*	3	*Pseudomonas veronii*
5	*Enterococcus faecium*	2	*Streptococcus equi*
5	*Enterococcus faecalis*	2	*Paenibacillus jamilae*
4	*Stenotrophomonas maltophilia*	2	*Kocuria rosea*
4	*Oligella ureolytica*	2	*Enterococcus gallinarum*
3	*Streptococcus thermophilus*	2	*Enterobacter hormaechei*
3	*Pseudomonas chlororaphis*	1	*Staphylococcus saprophyticus*
3	*Enterococcus camelliae*	1	*Shigella boydii*
2	*Streptococcus equi*	1	*Pseudomonas costantinii*
2	*Proteus hauseri*	83100	Total Reads
2	*Paenibacillus jamilae*		
2	*Lysinibacillus boronitolerans*		
2	*Haemophilus influenzae*		
2	*Haemophilus aegyptius*		
2	*Escherichia fergusonii*		
2	*Bacillus sonorensis*		
2	*Bacillus polygoni*		
2	*Bacillus luteolus*		
2	*Anoxybacillus kaynarcensis*		
1	*Virgibacillus kekensis*		
1	*Thermoleophilum minutum*		
1	*Staphylococcus saprophyticus*		
1	*Pseudomonas tolaasii*		
1	*Pseudoclavibacter faecalis*		
1	*Proteus penneri*		
1	*Pontibacillus halophilus*		
1	*Planifilum yunnanense*		
1	*Paenibacillus koleovorans*		
1	*Lysinibacillus sphaericus*		
1	*Kocuria kristinae*		
1	*Enterococcus durans*		
1	*Clostridium hydrogeniformans*		
1	*Bacillus thermoamylovorans*		
1	*Bacillus subtilis*		
1	*Bacillus seohaeanensis*		
1	*Bacillus locisalis*		

1	*Bacillus endoradicis*		
1	*Bacillus drentensis*		
1	*Bacillus beringensis*		
1	*Bacillus barbaricus*		
1	*Amphibacillus marinus*		
80377	Total Reads		

Organism sequenced: *Aggregatibacter aphrophilus* ATCC 33389			
blastn number of reads matched	blastn organism matched	SequenceMatch number of reads matched	SequenceMatch organism matched
59108	*Aggregatibacter aphrophilus*	71218	*Aggregatibacter aphrophilus*
18	*Klebsiella oxytoca*	21	*Haemophilus influenzae*
9	*Listeria monocytogenes*	20	*Listeria monocytogenes*
7	*Bacteroides ovatus*	20	*Klebsiella oxytoca*
5	*Shigella sonnei*	9	*Bacteroides ovatus*
4	*Shigella boydii*	7	*Corynebacterium striatum*
4	*Neisseria gonorrhoeae*	5	*Shigella sonnei*
4	*Enterococcus saccharolyticus*	5	*Proteus vulgaris*
4	*Corynebacterium simulans*	5	*Ochrobactrum anthropi*
3	*Stenotrophomonas maltophilia*	5	*Enterobacteriaceae bacterium*
3	*Staphylococcus epidermidis*	4	*Proteus mirabilis*
3	*Proteus vulgaris*	4	*Oligella ureolytica*
3	*Oligella ureolytica*	4	*Neisseria gonorrhoeae*
3	*Haemophilus aegyptius*	4	*Enterococcus saccharolyticus*
3	*Escherichia coli*	4	*Enterococcus faecalis*
3	*[Clostridium] sordellii*	4	*[Clostridium] sordellii*
2	*Streptococcus thermophilus*	3	*Stenotrophomonas maltophilia*
2	*Staphylococcus sciuri*	3	*Staphylococcus epidermidis*
2	*Staphylococcus aureus*	3	*Clostridium septicum*
2	*Shigella flexneri*	2	*Streptococcus salivarius*
2	*Pseudomonas aeruginosa*	2	*Staphylococcus sciuri*
2	*Proteus mirabilis*	2	*Staphylococcus aureus*
2	*Proteus hauseri*	2	*Pseudomonas aeruginosa*
2	*Ochrobactrum anthropi*	2	*Escherichia coli*
2	*Haemophilus influenzae*	2	*Enterobacter ludwigii*
2	*Enterococcus faecalis*	2	*Enterobacter hormaechei*
2	*Enterococcus camelliae*	2	*Eikenella corrodens*
2	*Eikenella corrodens*	2	*Clostridium perfringens*
2	*Clostridium septicum*	1	*Xanthomonadales bacterium*
1	*Streptococcus pyogenes*	1	*Streptococcus pyogenes*
1	*Staphylococcus saprophyticus*	1	*Streptococcus pneumoniae*
1	*Shigella dysenteriae*	1	*Staphylococcus saprophyticus*
1	*Pantoea vagans*	1	*Shigella boydii*
1	*Paenibacillus jamilae*	1	*Serratia plymuthica*
1	*Escherichia fergusonii*	1	*Paenibacillus polymyxa*

1	*Clostridium perfringens*	1	*Paenibacillus jamilae*
59217	Total Reads	71374	Total Reads
Organism sequenced: *Haemophilus influenzae* ATCC 9007			
blastn number of reads matched	blastn organism matched	SequenceMatch number of reads matched	SequenceMatch organism matched
17605	*Haemophilus influenzae*	58116	*Haemophilus influenzae*
16961	*Haemophilus aegyptius*	39	*Klebsiella oxytoca*
39	*Klebsiella oxytoca*	21	*Neisseria gonorrhoeae*
19	*Neisseria gonorrhoeae*	14	*Aggregatibacter aphrophilus*
9	*Listeria monocytogenes*	13	*Listeria monocytogenes*
9	*Aggregatibacter aphrophilus*	9	*Proteus mirabilis*
8	*Staphylococcus sciuri*	9	*Ochrobactrum anthropi*
7	*Shigella sonnei*	8	*Staphylococcus sciuri*
7	*Pseudomonas aeruginosa*	8	*Shigella sonnei*
7	*Proteus mirabilis*	8	*Pseudomonas aeruginosa*
7	*Ochrobactrum anthropi*	7	*Bacteroides ovatus*
6	*Oligella ureolytica*	6	*Proteus vulgaris*
4	*Staphylococcus aureus*	6	*Oligella ureolytica*
4	*Proteus vulgaris*	6	*Escherichia coli*
4	*Enterococcus saccharolyticus*	5	*Haemophilus haemolyticus*
4	*Clostridium septicum*	5	*[Clostridium] sordellii*
4	*Bacteroides ovatus*	5	*Clostridium septicum*
4	*Avibacterium endocarditidis*	4	*Staphylococcus aureus*
3	*Stenotrophomonas maltophilia*	4	*Enterococcus saccharolyticus*
3	*Shigella flexneri*	4	*Enterococcus faecium*
3	*Shigella dysenteriae*	4	*Eikenella corrodens*
3	*Shigella boydii*	4	*Corynebacterium striatum*
3	*Enterococcus faecium*	3	*Streptococcus pneumoniae*
3	*Corynebacterium simulans*	3	*Stenotrophomonas maltophilia*
3	*[Clostridium] sordellii*	3	*Citrobacter freundii*
2	*Streptococcus pyogenes*	2	*Streptococcus pyogenes*
2	*Streptococcus pneumoniae*	2	*Shigella flexneri*
2	*Elizabethkingia meningoseptica*	2	*Haemophilus aegyptius*
2	*Eikenella corrodens*	2	*Enterobacteriaceae bacterium*
1	*Streptococcus equi*	2	*Elizabethkingia meningoseptica*
1	*Staphylococcus saprophyticus*	1	*Streptococcus equi*
1	*Proteus hauseri*	1	*Staphylococcus saprophyticus*
1	*Kocuria kristinae*	1	*Shigella boydii*
1	*Enterococcus casseliflavus*	1	*Paenibacillus polymyxa*
1	*Enterococcus camelliae*	1	*Kocuria rosea*
34743	Total Reads	1	*Escherichia fergusonii*
		1	*Enterococcus gallinarum*
		1	*Enterococcus casseliflavus*
		1	*Enterobacter ludwigii*

		1	*Enterobacter hormaechei*
		1	*Clostridium perfringens*
		58335	Total Reads
Organism sequenced: *Neisseria gonorrhoeae* ATCC 19424			
blastn number of reads matched	blastn organism matched	SequenceMatch number of reads matched	SequenceMatch organism matched
116113	*Neisseria gonorrhoeae*	126608	*Neisseria gonorrhoeae*
25	*Klebsiella oxytoca*	26	*Klebsiella oxytoca*
16	*Shigella sonnei*	18	*Listeria monocytogenes*
13	*Listeria monocytogenes*	11	*Eikenella corrodens*
8	*Eikenella corrodens*	10	*Oligella ureolytica*
7	*Proteus vulgaris*	9	*Stenotrophomonas maltophilia*
7	*Proteus mirabilis*	9	*Proteus mirabilis*
7	*Ochrobactrum anthropi*	9	*Bacteroides ovatus*
7	*Bacteroides ovatus*	8	*Ochrobactrum anthropi*
7	*Aggregatibacter aphrophilus*	8	*Enterococcus saccharolyticus*
6	*Pseudomonas aeruginosa*	8	*Enterobacteriaceae bacterium*
6	*Oligella ureolytica*	8	*Corynebacterium striatum*
6	*Enterococcus saccharolyticus*	8	*Aggregatibacter aphrophilus*
6	*Corynebacterium simulans*	7	*Shigella sonnei*
5	*Streptococcus pneumoniae*	7	*Proteus vulgaris*
4	*Stenotrophomonas maltophilia*	6	*Streptococcus pyogenes*
4	*Enterococcus casseliflavus*	6	*Streptococcus pneumoniae*
3	*Streptococcus thermophilus*	6	*Pseudomonas aeruginosa*
3	*Streptococcus pyogenes*	6	*Haemophilus influenzae*
3	*Staphylococcus aureus*	5	*Escherichia coli*
3	*Clostridium septicum*	4	*Enterococcus casseliflavus*
2	*Streptococcus equi*	3	*Streptococcus salivarius*
2	*Shigella flexneri*	3	*Staphylococcus aureus*
2	*Shigella dysenteriae*	3	*Paenibacillus polymyxa*
2	*Paenibacillus polymyxa*	3	*Clostridium septicum*
1	*Staphylococcus sciuri*	2	*Streptococcus equi*
1	*Staphylococcus saprophyticus*	2	*Staphylococcus sciuri*
1	*Staphylococcus epidermidis*	2	*Enterobacter hormaechei*
1	*Shigella boydii*	2	*[Clostridium] sordellii*
1	*Proteus hauseri*	2	*Citrobacter freundii*
1	*Haemophilus influenzae*	1	*Staphylococcus saprophyticus*
1	*Enterococcus faecium*	1	*Staphylococcus epidermidis*
1	*Enterococcus faecalis*	1	*Shigella flexneri*
1	*Enterococcus camelliae*	1	*Enterococcus gallinarum*
1	*Elizabethkingia meningoseptica*	1	*Enterococcus faecium*
1	*Clostridium perfringens*	1	*Enterococcus faecalis*
1	*Citrobacter koseri*	1	*Elizabethkingia meningoseptica*
1	*Bacillus vietnamensis*	1	*Clostridium perfringens*

116280	Total Reads	126817	Total Reads
